# Genome-Wide Association Mapping for Yield and Related Traits Under Drought Stressed and Non-stressed Environments in Wheat

**DOI:** 10.3389/fgene.2021.649988

**Published:** 2021-06-22

**Authors:** S. M. Hisam A. Rabbi, Ajay Kumar, Sepehr Mohajeri Naraghi, Senay Simsek, Suraj Sapkota, Shyam Solanki, Mohammed S. Alamri, Elias M. Elias, Shahryar Kianian, Ali Missaoui, Mohamed Mergoum

**Affiliations:** ^1^Department of Plant Sciences, North Dakota State University, Fargo, ND, United States; ^2^Institute of Plant Breeding, Genetics, and Genomics, University of Georgia, Griffin, GA, United States; ^3^Department of Crop and Soil Sciences, Washington State University, Pullman, WA, United States; ^4^Department of Food Sciences and Nutrition, King Saud University, Riyadh, Saudi Arabia; ^5^United States Department of Agriculture-The Agricultural Research Service (USDA-ARS) Cereal Disease Laboratory, University of Minnesota, St. Paul, MN, United States; ^6^Department of Crop and Soil Sciences, University of Georgia, Griffin, GA, United States

**Keywords:** drought tolerance, hard red spring wheat, association mapping, quantitative trait loci, marker-assisted selection

## Abstract

Understanding the genetics of drought tolerance in hard red spring wheat (HRSW) in northern USA is a prerequisite for developing drought-tolerant cultivars for this region. An association mapping (AM) study for drought tolerance in spring wheat in northern USA was undertaken using 361 wheat genotypes and Infinium 90K single-nucleotide polymorphism (SNP) assay. The genotypes were evaluated in nine different locations of North Dakota (ND) for plant height (PH), days to heading (DH), yield (YLD), test weight (TW), and thousand kernel weight (TKW) under rain-fed conditions. Rainfall data and soil type of the locations were used to assess drought conditions. A mixed linear model (MLM), which accounts for population structure and kinship (PC+K), was used for marker–trait association. A total of 69 consistent QTL involved with drought tolerance-related traits were identified, with *p* ≤ 0.001. Chromosomes 1A, 3A, 3B, 4B, 4D, 5B, 6A, and 6B were identified to harbor major QTL for drought tolerance. Six potential novel QTL were identified on chromosomes 3D, 4A, 5B, 7A, and 7B. The novel QTL were identified for DH, PH, and TKW. The findings of this study can be used in marker-assisted selection (MAS) for drought-tolerance breeding in spring wheat.

## Introduction

Wheat (*Triticum aestivum* L.) is a major crop worldwide contributing about 20% of calories to the human population. Current genetic and genomic improvements in wheat have helped increase its production; however, further improvements are essential to increasing wheat productivity to feed the world's population, which is projected to reach over nine billion by 2050 (Hertel, 2011; Sapkota et al., 2019). Wheat production is often reduced by several biotic and abiotic stresses including drought and heat. Plant breeding has improved crop resistance to both biotic and abiotic stresses; nevertheless, the progress is slow and the yield gap between stress-prone areas and favorable production regions of major crops, including wheat, is high (Edae et al., 2014). Therefore, breeding efforts are being focused on dissecting the genetics of abiotic stresses including drought in wheat to develop knowledge and resources to speed up the development of climate-resilient wheat cultivars.

Drought poses a major threat to crop yield, highlighting the urgent need to develop drought-tolerant cultivars (Ergen and Budak, 2009). The majority of countries worldwide experience drought problems, even those in humid regions as they often have dry spells at some point. Obviously, drought is more severe in arid areas with minimal rainfall (Sun et al., 2006). North Dakota is the biggest producer of hard-red spring wheat (HRSW) in the USA (North Dakota Wheat Commission, 2016). The state, especially the semiarid western half, experiences frequent droughts (Climate Change and the Economy, 2008). Consequently, HRSW, a major cash crop for ND and the USA, is regularly affected by drought in this region. Developing and releasing drought-tolerant HRSW cultivars is critical to countering ND drought conditions, but this cannot be done without understanding the genetics of drought tolerance for HRSW in northern USA.

Quantitative trait locus (QTL) analysis allows genetic dissection, which can be a sound approach for understanding the molecular basis of drought tolerance in HRSW. In the past, several QTL mapping studies for drought tolerance in wheat were conducted (Kirigwi et al., 2007; Alexander et al., 2012; Ibrahim et al., 2012a,b). These studies have used different types of markers, including SSRs, EST-STS, and DArTs. However, almost all of these studies were based on low-resolution molecular maps consisting of only 102–690 markers. The number of markers in the previous studies seems insufficient to saturate the wheat genome due to its large size of 17 gigabase pairs (Brenchley et al., 2012). Also, drought tolerance is a quantitative trait adopting different mechanisms (Blum, 1988) and should have several QTL distributed throughout the whole genome. A high-resolution map can provide a more complete genetic dissection of drought tolerance and also a successful application of associated molecular markers through marker-assisted selection (MAS) programs. The Infinium iSelect 90K assay (Wang et al., 2014), with more than 81,000 gene-associated SNPs to assess polymorphism in bread wheat, provides a better means to identify SNPs tightly linked to drought tolerance.

Biparental QTL mapping, even when using high-density linkage maps, suffers some limitations. The biparental population has fewer recombination events and therefore has low resolution. By comparison, association mapping (AM) exploits a broader population and multiple alleles and has a better resolution of the QTL (Yu and Buckler, 2006). A few AM studies on drought tolerance conducted in the past have used a small number of markers (Dodig et al., 2012; Edae et al., 2013, 2014; Ballesta et al., 2020; Maulana et al., 2020), which seems insufficient to explore the variation in wheat, efficiently. Dodig et al. (2012) used 46 SSR markers, and Edae et al. (2013) used 78 DArT markers. Maulana et al. (2020) used greenhouse for phenotyping for the association study, whereas Ballesta et al. (2020) used field experiments for their study but showed the association only for chromosome 4A. Furthermore, to our knowledge, no genetic studies were conducted on HRSW germplasm to elucidate the QTL associated with drought-related traits in the region. Therefore, the present study was undertaken to identify genes/QTL associated with yield and agronomic traits evaluated under drought conditions in field experiments using an association mapping approach combined with high-density SNP marker assay.

## Materials and Methods

### Plant Materials

In 2012, a germplasm panel comprising of 350 HRSW inbred lines developed by the HRSW breeding program at North Dakota State University (NDSU) and different cultivars with varying drought tolerances was used for this study (Supplementary Table 1). Eleven more accessions were added for the experiments conducted in 2013 and 2014 (Supplementary Table 2). These lines were developed over time from different crosses, and pedigree selections for different purposes such as drought tolerance, disease resistance, quality, and yield were used for this study.

### Field Experiments and Data Collection

The evaluation of agronomic performances of the AM panel was carried out under non-irrigated field conditions at different locations in ND, USA. In 2012, the AM was evaluated at Prosper (46.96300°N, 97.01980°W), Casselton (46.540N, 97.1238°W), and Minot (48°13′59″N 101°17′32″W). In 2013, the evaluation was carried out in Prosper, Minot, and Williston (48°9′23″N 103°37′41″W), while in 2014, it was evaluated in Prosper, Minot, and Hettinger (46°0′3″N 102°38′0″W). Prosper and Casselton are located in eastern ND, at 46.9630° N, 97.0198° W, and 46.9° N, 97.210556° W, respectively. Minot is situated between western ND's semiarid grassland and central ND's subhumid grassland (48.2330° N, 101.2923° W). Williston's location is in northwestern ND (48.1470° N, 103.6180° W), and Hettinger's is in southwestern ND (46.0014° N, 102.6368° W). The total rainfall during the growing period (seed sowing to ripening) in 2012, 2013, and 2014 at Prosper was 119.6, 269.7, and 168.6 mm, respectively (Table 1). Minot's rainfall during the growing period was 168 mm in 2012, 159.8 mm in 2013, and 230.9 mm in 2014. In case of Casselton, Williston, and Hettinger, the rainfall was 122.8 mm (2012), 319.3 mm (2013), and 200.3 mm (2014), respectively, during the growing season (North Dakota Agricultural Weather Network, 2015). The total rainfall in the experimental sites during the growing period was considered to assess the drought condition (Table 1). The water holding capacity of the experimental sites was achieved from the soil type (Frazen, 2003). In 2012, the experiment was conducted in a randomized complete block design (RCBD) with two replicates, whereas a simple lattice design was used in 2013 and 2014. The plots had an area of 2.44 × 1.22 m and seven rows with a 15.24 cm gap between them in 2012 and 2013. The plot size in 2014 was 2.44 × 1.42 m, but the number of rows was still seven with a larger gap (17.78 cm) between them.

**Table 1:** Soil types, plant-available water (water-holding capacity of soil), and total rainfall for nine environments used in this study.

**Environments**	**Soil type**	**Plant-available water (mm water/30.48 cm soil)**	**Rainfall (mm)**
Casselton 2012	Fine silty loam	45.72–63.5	120.1
Prosper 2012	Fine silty loam	45.72–63.5	119.6
Minot 2012	Fine sandy loam	31.75–45.72	168
Prosper 2013	Fine silty loam	45.72–63.5	269.7
Minot 2013	Fine sandy loam	31.75–45.72	442.3
Williston 2013	Fine sandy loam	31.75–45.72	319.3
Minot 2014	Fine sandy loam	31.75–45.72	230.9
Prosper 2014	Fine silty loam	45.72–63.5	168.6
Hettinger 2014	Fine sandy loam	31.75–45.72	200.3

The phenotypic data was collected on DH, PH, YLD, TW, and TKW. The heading date (DH) was recorded when more than 50% of the plants in the plot were starting to flower. Plant height (PH) was measured in the middle of the plot from plant base to tip excluding the awn. Yield per plot (YLD) was converted to yield/ha for further analysis. Similarly, kg/0.5-pint cup was converted to kg/m^3^ as the test weight (TW) for further analysis. A thousand kernels were counted using a seed counter and were weighed for thousand kernel weight (TKW).

### Single-Nucleotide Polymorphism Genotyping

Genomic DNA was isolated from lyophilized young leaves of each genotype using the DNeasy Plant Mini Kit (Qiagen, Valencia, CA, cat. no. 69106). The quality of the DNA was checked on 0.8% agarose gel. The NanoDrop 1000 spectrophotometer (NanoDrop Technologies Inc., Wilmington, DE) was used to check the DNA concentration. The accessions of the AM panel were genotyped using the Illumina 90K iSelect wheat SNP assay (Wang et al., 2014) in the Small Grains Genotyping Lab at USDA-ARS, Fargo, ND. The Illumina iSelect 90K assay produced data for 81,587 SNPs. The analyses of SNP genotyping, clustering of the SNP alleles, and calling of the genotypes were performed with Genome Studio v2011.1 (www.illumina.com). The minimum number of points used in the cluster was 10 (Wang et al., 2014). Monomorphic SNPs and SNPs having more than 20% missing genotypic data and 10% heterozygosity were excluded. The best linear unbiased prediction (iBLUP) method (Yang et al., 2014b) was used to impute the missing genotypic data for the remaining SNPs. The polymorphic SNPs selected after filtering based on the above mentioned criteria were screened for their positions on the chromosomes based on the wheat consensus genetic map (Wang et al., 2014).

### Phenotypic Data Analysis (ANOVA, Descriptive Statistics, and Frequency Distribution)

The ANOVA Proc MIXED procedure was used (SAS Institute, 2004) to analyze the phenotypic data from 2012, whereas for 2013 and 2014, the Proc LATTICE was used. The accessions of the AM panel were considered as fixed effects, and environments and blocks were considered as random effects in the ANOVA Proc MIXED procedure. The mean values were separated using the *F*-protected least significant difference (LSD) value at the *P* ≤ 0.05 level of significance. CORR procedure of SAS (SAS Institute, 2004) was used to calculate Pearson correlations between traits for each environment. The phenotypic data with a low coefficient of variance (CV) value and significant differences among entries were used for further analysis. The locations that did not show significant differences for most of the traits and with a high CV were not included for further analysis and reporting.

### Marker–Trait Association Analysis

Population structure was calculated using markers with pairwise *R*^2^ < 0.5 for all pairwise comparisons. To assign the subpopulation membership for each genotype, STRUCTURE software version 3.2 was used (Pritchard et al., 2000). We used an admixture model with independent allele frequencies, a burn-in of 100,000, and an MCMC replication of 500,000 for *K* = 1–10 with five replications. The delta k calculated from the STRUCTURE software was used to select the optimum number of subpopulations. The number of subpopulations (k) was plotted against the delta k calculated using the STRUCTURE software. Pairwise linkage disequilibrium (LD) between markers in the null model was calculated as the squared allele frequency correlation (*R*^2^) in the R-package (Lipka et al., 2012) after filtering for minor allele frequency (MAF) ≥ 5%. Genome-wide LD decay was estimated by plotting *R*^2^ against the corresponding pairwise genetic distance (cM) (Wang et al., 2014). AM analysis was conducted using the software TASSEL v.5.0 (Bradbury et al., 2007). The mixed linear model (MLM) with PC + Kinship (K) was used for AM, where the genotypic data were filtered for minor allele (≤ 5%) frequency. A total of 14,816 filtered SNPs were used for further AM study. The initial cutoff point for marker–trait association (MTA) was considered at *p* ≤ 0.001. Then, this cutoff was subjected to Bonferroni correction (Yang et al., 2014a) to get the threshold (*p* ≤ 3.4 ^*^ 10^−6^). Only the markers identified to be associated in at least two environments were reported.

### Candidate Gene Analysis

For candidate gene analyses, the sequences of the markers showing MTAs were obtained from the T3/Wheat database (Blake et al., 2016) and their physical positions were extracted using the BLAST search against Chinese_Spring_IWGSC_RefSeq1.0 (Appels et al., 2018, Alaux et al., 2018) to identify the most proximal gene. The physical position of each marker was utilized to identify if it represents the perfect marker in the annotated gene space of Chinese Spring. For markers anchored in the non-gene space, flanking genes were obtained by manual IWGSC RefSeq1.0 (Blake et al., 2019) genome scanning in the GrainGenes Genome Browser (https://wheat.pw.usda.gov/GG3/genome_browser). For each MTA, the linked gene set can be extracted from the GrainGenes database. Finally, annotation of identified genes was included to predict their function. *In silico* expression analysis was carried out in the Wheat Expression Browser expVIP (Ramírez-González et al., 2018, Borrill et al., 2016) dataset for drought and heat stress and PEG to stimulate drought.

## Results

### Phenotypic Analyses

**Figure 1:**
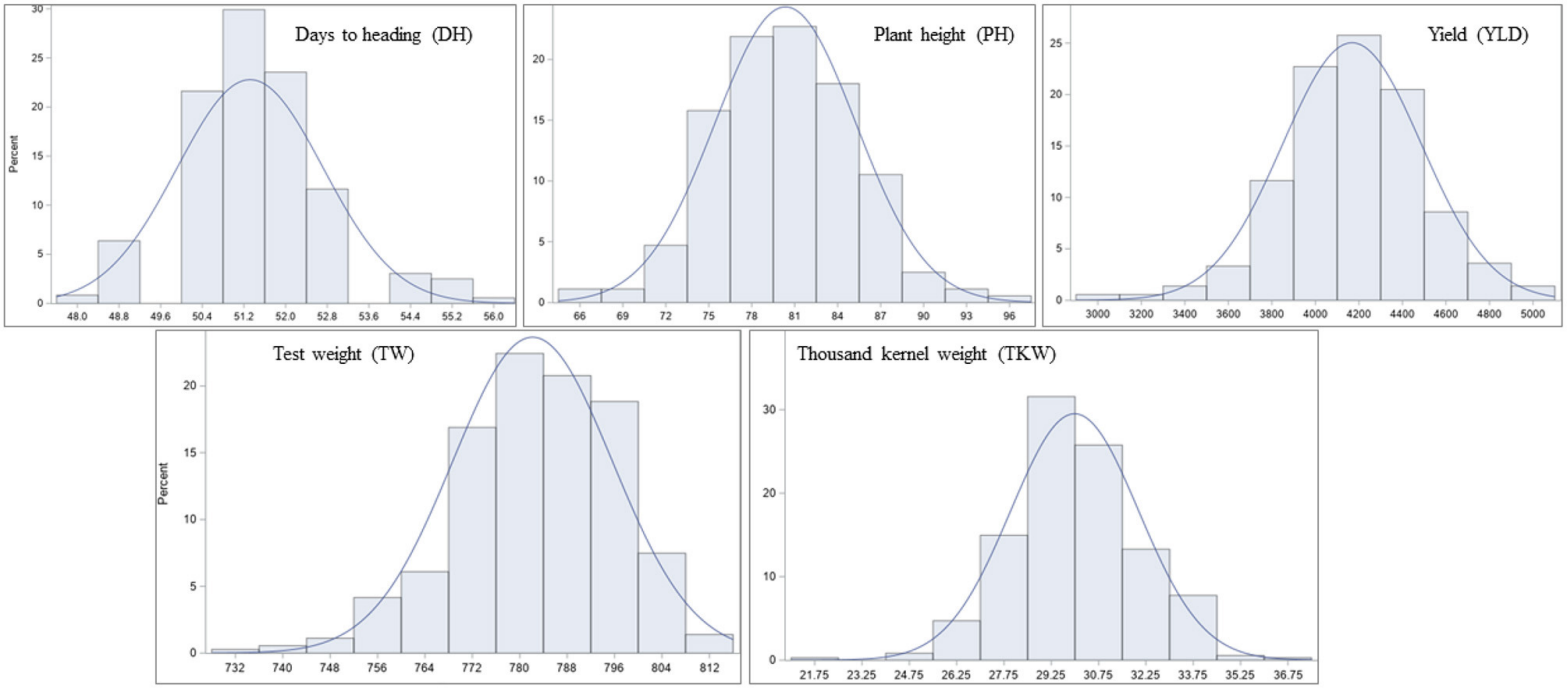
Frequency distribution of least-square means (lsmeans) for the agronomic traits in the hard red spring wheat panel across environments. The panel was evaluated for days to heading (DH), plant height (PH), yield (YLD), test weight (TW), and thousand kernel weight (TKW) in nine different environments of North Dakota in 2012, 2013, and 2014.

Significant differences among genotypes were found in the environments of Casselton 2012, Prosper 2012, Minot 2012, Prosper 2013, Prosper and Minot 2013 and 2014, and Hettinger 2014 (Table 2). The rest of the phenotypic data was not analyzed further. The seeds of Minot 2013 could not be cleaned due to Fusarium head blight infection, and hence, YLD, TW, and TKW could not be reported for that environment. Also, TKW for Minot 2014 and Hettinger 2014 was not reported. The phenotypic analyses of the data showed that the heading date had a highly significant negative correlation with YLD, TW, and TKW in all environments (Table 2). Heading date showed a significant positive correlation with PH in four environments. Plant height had a significantly negative correlation with YLD in four environments including the overall mean. It had a significantly positive correlation with TW in six environments, whereas it did not show any correlation with TKW. The yield had a strong positive association with TW in five environments. Also, it showed a strong positive association with TKW in all the environments including the overall mean. Again, TW had a strong positive correlation with TKW in three environments (Table 2). The frequency distributions of the phenotypic data for DH, PH, YLD, TW, and TKW showed a continuous distribution, a characteristic of the typical quantitative traits (Figure 1).

**Table 2:** Correlation coefficients between five agronomic traits in the association mapping panel in different environments (Env.) and overall mean across environments (M).

**Trait ᵻ and Env ǂ**	**PH**	**DH**	**YLD**	**TW**
**DH**				
1	0.16^**^			
2	0.07*ns*			
3	0.15^**^			
4	0.18^***^			
5	0.06*ns*			
6	0.09*ns*			
7	0.09^*^			
8	0.09*ns*			
**YLD**				
1	−0.13^*^	−0.21^***^		
2	0.04*ns*	−0.4^***^		
3	0.13^*^	−0.16^**^		
4	0.13^*^	0.20^***^		
5	−0.16^**^	−0.15^**^		
6	−0.13^*^	−0.15^**^		
7	.	.		
8	−0.36^***^	−0.24^***^		
**TW**				
1	0.24^***^	−0.16^**^	0.18^**^	
2	0.27^***^	−0.35^***^	0.41^***^	
3	0.22^**^	−0.27^***^	0.42^***^	
4	0.12^*^	−0.03*ns*	−0.03*ns*	
5	0.09*ns*	−0.17^**^	0.30^***^	
6	0.25^***^	−0.34^***^	0.24^***^	
7	.	.	.	
8	0.2^***^	−0.27^***^	0.08*ns*	
**TKW**				
1	0.04*ns*	−0.14^**^	0.27^***^	0.11^*^
2	0.04*ns*	−0.15^**^	0.32^***^	0.19^***^
3	0.13^*^	−0.21^***^	0.29^***^	0.39^***^
4	0.05*ns*	0.05*ns*	0.30^***^	−0.09*ns*
5	.	.	.	.
6	.	.	.	.
7	.	.	.	.
8	−0.01*ns*	−0.10*ns*	0.34^***^	0.10*ns*

* *Significant at p < 0.05*,

** *Significant at p < 0.01*,

*** *Significant at p < 0.001 level*;

*ᵻPH, Plant height; DH, Days to heading; YLD, Yield; TW, Test weight; TKW, Thousand kernel weightǂ1 = Casselton 2012, 2 = Prosper 2012, 3 = Minot 2012, 4 = Prosper 2013, 5 = Prosper 2014, 6 Hettinger 2014, 7 = Minot 2013, 8 = Mean across environments*.

### Marker Distribution, Population Structure, and LD

**Figure 2:**
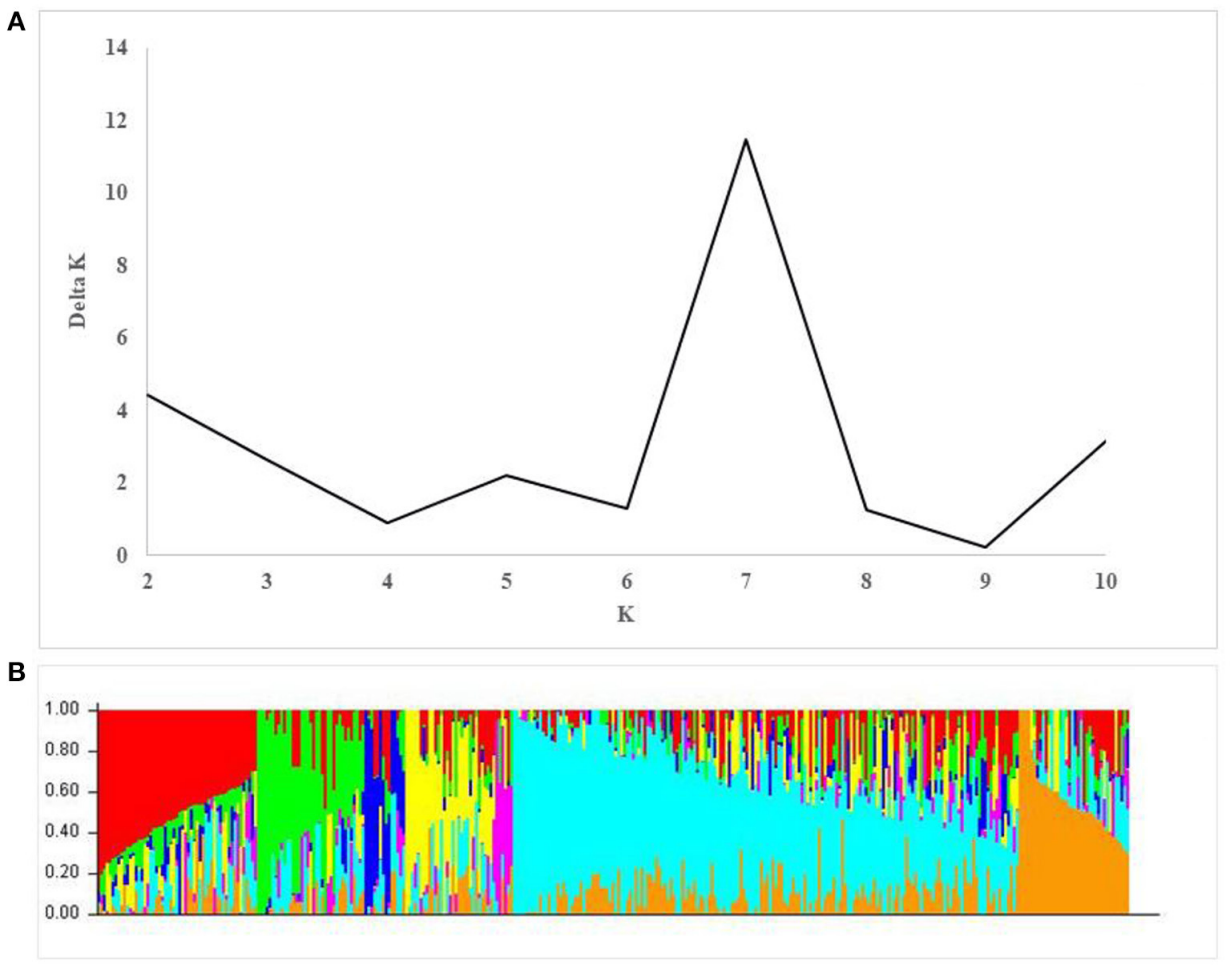
Population structure in the association mapping (AM) panel. **(A)** Estimation of the number of populations by calculating delta K values. **(B)** Estimated population structure of the AM panel (*k* = 7).

A total of 17,514 polymorphic SNPs were selected after the filtering-based criteria mentioned earlier in the material and methods section (Supplementary Table 3). An additional 2,756 SNPs were excluded for lacking map positions on the consensus hexaploid wheat maps (Wang et al., 2014). Out of 14,816 SNP markers used in the AM study, 7,848 were located on the B-genome, 5,503 on the A-genome, and 1,465 markers on the D-genome. The D-genome had the lowest density of markers, with an average distance of 0.87 cM between two markers. The number of markers on individual chromosomes ranged from 56 (4D) to 1,433 (2B). The average number of markers per chromosome was 705.52 (Table 3). The number of subpopulations (k) was plotted against the delta k calculated using software STRUCTURE. The peak of the broken line graph was observed at *k* = 7, indicating that the natural population can be divided into seven subpopulations (Figure 2). The association was analyzed using five principal components (PC), which captured 25% of the variation. Genome-wide LD decay was estimated by plotting the *R*^2^ value against the corresponding pairwise genetic distance (cM) and the LD heat map was created for the whole genome (Supplementary Figure 1). The LD pattern varied by chromosome even after controlling for population relatedness. Overall, the A and B genomes showed high LD compared to the D-genome. The LD dropped at an approximate genetic distance of 10 cM, and therefore, ±10 cM was used to establish confidence intervals for QTL regions. Furthermore, SNP markers with a pairwise *R*^2^ ≥ 0.7 were considered as a single locus.

**Table 3:** Chromosome and genome wide distribution of markers in our spring wheat association mapping panel based on the 90k SNP consensus map (Wang et al., 2014).

**Chromosome**	**No. of markers**	**Map length**	**Average map density**
			**cM/marker**
1A	785	156.3	0.2
2A	861	185.47	0.22
3A	661	197.2	0.3
4A	663	166.71	0.25
5A	783	148.3	0.19
6A	852	175.32	0.21
7A	898	244.16	0.28
1B	1,197	173.62	0.15
2B	1,433	188.27	0.13
3B	1,139	154.48	0.14
4B	635	118.91	0.19
5B	1,348	219.77	0.16
6B	1,216	122.92	0.1
7B	880	188.64	0.21
1D	261	199.86	0.77
2D	476	152.84	0.32
3D	207	152.84	0.74
4D	56	170.43	3.04
5D	147	207.33	1.41
6D	170	160.5	0.94
7D	148	226.87	1.53
A genome	5,503	1,273.46	0.23
B genome	7,848	1,166.61	0.15
D genome	1,465	1,270.67	0.87
Whole genome	14,816	3,710.74	0.25

### Identification of QTL and Associated Candidate Genes

In this study, we detected a total of 69 QTL involved with drought tolerance on all chromosomes except 5D using AM (Table 4; Supplementary Figure 2). Twenty QTL six were associated with DH. These QTL explained 5.6–11.33% of phenotypic variation (PV). Five of those QTL explained >10% of PV and therefore were considered major QTL. Twelve of the QTL were identified to be constitutive, and eight of the QTL were identified exclusively in drought-prone environments. Similarly, a total of 20 QTL six were associated with PH. These QTL explained 4.54–48.01% of PV, with major effects (>10% PV). Sixteen QTL were identified as constitutive, three were identified in the control environments, and one was identified in the drought environment (Table 4).

**Table 4:** Marker-trait associations (QTL) identified for yield and related traits under different environments in this study.

**QTL and trait**	**QTL region**	**Other associated traits ᵻ**	**Env. ǂ**	**Water regime**	**Position^§^**	***p*-value^**¶**^**	***R^**2**^* (%)**
**Days to heading**							
*QDH.ndsu.1B*	5		1^*^, 2, 4^*^	Control+drought	90.26	4.83^*^10^−7^	8.96
*QDH.ndsu.2A.2*	10		1^*^, 2, 4^*^	Control+drought	113.30	1.92^*^10^−6^	8.07
*QDH.ndsu.2B.2*	14	PH, TKW	1^*^, 2, 4^*^, 6^*^, 7^*^	Control+drought	99.8–104.39	4.86^*^10^−8^	10.44
*QDH.ndsu.2D*	16		2^*^, 4	Control+drought	19.03	2.98^*^10^−6^	7.54
*QDH.ndsu.3A.1*	17	PH, TW	1^*^, 2, 6^*^, 7^*^	Control+drought	90.55	1.25^*^10^−8^	11.33
*QDH.ndsu.3B*	21	TW, YLD	1^*^, 2, 3^*^, 4^*^, 5^*^	Control+drought	70.09	1.44^*^10^−8^	11.24
*QDH.ndsu.4A.1*	25	TKW	1^*^, 2, 3^*^, 4^*^	Control+drought	51.97	1.83^*^10^−6^	8.10
*QDH.ndsu.4B*	28	TW, PH, YLD	1^*^, 2^*^, 4^*^, 7^*^	Control+drought	64.03–75.64	7.35^*^10^−6^	7.22
*QDH.ndsu.4D*	29		1^*^, 2, 4^*^	Control+drought	94.22	3.11^*^10^−8^	10.74
*QDH.ndsu.5B.2*	35		1^*^, 2^*^, 3^*^, 4^*^, 5^*^	Control+drought	100.64–110.19	7.67^*^10^−6^	7.19
*QDH.ndsu.6B*	39	TKW, PH, YLD	1^*^, 2^*^, 6^*^, 7^*^	Control+drought	63.14–71.76	2.97^*^10^−5^	6.34
*QDH.ndsu.7B*	44	YLD	2^*^, 3^*^, 4^*^	Control+drought	98.30–101.18	9.18^*^10^−5^	5.60
*QDH.ndsu.2A.1*	8	TW	1^*^, 2	Drought	25.02	4.95^*^10^−8^	10.43
*QDH.ndsu.2A.3*	11	YLD	1^*^, 2	Drought	141.66	1.81^*^10^−6^	8.11
*QDH.ndsu.2B.1*	13	YLD	1^*^, 2	Drought	83.80	8.13^*^10^−7^	8.62
*QDH.ndsu.3A.2*	18	YLD	1^*^, 2	Drought	117.73	1.58^*^10^−7^	9.68
*QDH.ndsu.4A.2*	26	TKW	1^*^, 2^*^, 3^*^	Drought	99.19–103.03	3.69^*^10^−5^	6.21
*QDH.ndsu.5A.1*	30		1^*^, 2	Drought	55.01	2.54^*^10^−6^	7.90
*QDH.ndsu.5A.2*	31		1^*^, 2^*^, 3^*^	Drought	84.13	3.98^*^10^−6^	7.61
*QDH.ndsu.5B.1*	33	TKW	1^*^, 2	Drought	5.70	8.35^*^10^−7^	8.61
**Plant height**							
*QPH.ndsu.1A*	3		1^*^, 5, 6^*^	Control+drought	105.74	9.80^*^10^−13^	16.83
*QPH.ndsu.1B*	4	YLD	2^*^, 4^*^, 7	Control+drought	76.89	3.2^*^10^−7^	7.73
*QPH.ndsu.2A.1*	9		2^*^, 3^*^, 4^*^, 7	Control+drought	98.43–101.97	2.03^*^10^−5^	6.31
*QPH.ndsu.2A.2*	12		2^*^, 6^*^,7^*^	Control+drought	156.23–162.89	2.4^*^10^−5^	6.25
*QPH.ndsu.2B*	14	TKW, DH	2^*^, 3^*^, 4^*^, 5, 7^*^	Control+drought	109.53	2.24^*^10^−7^	8.99
*QPH.ndsu.3A.1*	17	TW, DH	2^*^, 3^*^, 4^*^	Control+drought	77.57	1.68^*^10^−4^	5.13
*QPH.ndsu.3A.2*	19		2^*^, 6	Control+drought	128.64	6.08^*^10^−7^	8.46
*QPH.ndsu.3A.3*	20		3^*^, 4^*^, 6^*^	Control+droughte	180.33	5.42^*^10^−5^	5.72
*QPH.ndsu.3B*	22		2^*^, 3^*^, 5^*^	Control+drought	102.54–106.73	5.29^*^10^−4^	4.54
*QPH.ndsu.3D.1*	23		2^*^, 5^*^, 7^*^	Control+drought	0–4.46	3.29^*^10^−4^	4.67
*QPH.ndsu.3D.2*	24		2^*^, 3^*^, 4^*^	Control+drought	66.99	1.62^*^10^−4^	5.27
*QPH.ndsu.4B*	28	TW, YLD, DH	1, 2, 3, 4, 5^*^, 6, 7^*^	Control+drought	56.19	3.79^*^10^−14^	19.97
*QPH.ndsu.5B*	34	YLD	2^*^, 3^*^, 4^*^,5, 6, 7^*^	Control+drought	63.07	6.51^*^10^−31^	48.01
*QPH.ndsu.6A.2*	38		1, 5^*^	Control+drought	133.74	8.66^*^10^−8^	10.07
*QPH.ndsu.6B.1*	39	TKW, DH, YLD	3^*^, 4^*^, 5, 6^*^, 7	Control+drought	56.98	1.8^*^10^−30^	47.15
*QPH.ndsu.7A.2*	43		2^*^, 3^*^, 4^*^	Control+drought	212.66	4.3^*^10^−6^	7.55
*QPH.ndsu.6A.1*	37		4, 5	Control	82.38	4.61^*^10^−10^	12.85
*QPH.ndsu.6B.2*	40	YLD	5, 7^*^	Control	108.86	1.07^*^10^−30^	47.60
*QPH.ndsu.6D*	41		4^*^, 5, 6^*^, 7^*^	Control	22.92	1.56^*^10^−7^	9.21
*QPH.ndsu.7A.1*	42		1^*^, 2 ^*^, 3^*^	Drought	61.36	3.38^*^10^−4^	4.83
**Thousand kernel weight**							
*QTKW.ndsu.2B.1*	14	PH, DH	1^*^, 2^*^, 3^*^	Drought	106.56–114.57	7.44^*^10^−5^	5.64
*QTKW.ndsu.2B.2*	15	YLD	2^*^, 3	Drought	155.41	9.33^*^10^−7^	8.55
*QTKW.ndsu.4A.1*	25	DH	1^*^, 2^*^, 3^*^	Drought	48.98–51.97	1.74^*^10^−4^	5.22
*QTKW.ndsu.4A.2*	26	DH	1^*^, 2^*^, 3^*^	Drought	105.87–108.72	1^*^10^−4^	5.59
*QTKW.ndsu.4A.3*	27		2, 3^*^	Drought	154.30	2.44^*^10^−7^	9.20
*QTKW.ndsu.6B*	39	PH, DH, YLD	1^*^, 2^*^, 3^*^	Drought	56.64–64.82	1.79^*^10^−4^	5.20
*QTKW.ndsu.5B*	33	DH	1^*^, 3, 4^*^	Control+drought	17.48	1.98^*^10^−6^	6.92
**Test weight**							
*QTW.ndsu.1A*	1		1^*^, 2^*^, 6^*^	Control+drought	29.11–38.11	4.39^*^10^−4^	4.63
*QTW.ndsu.2A*	8	DH	1^*^, 4^*^, 5^*^	Control+drought	20.26	7.12^*^10^−4^	4.07
*QTW.ndsu.3A*	17	PH, DH	1^*^, 2^*^, 5^*^	Control+drought	85.73	284^*^10^−4^	3.70
*QTW.ndsu.3B*	21	DH, YLD	1^*^, 3^*^, 4^*^, 5^*^	Control+drought	62.31–69.53	3.83^*^10^−6^	7.58
*QTW.ndsu.4B*	28	PH, YLD, DH	1, 2^*^, 3, 4^*^, 5^*^, 6^*^	Control+drought	55.55	4.66^*^10^−7^	7.66
**Yield**							
*QYL.ndsu.1A*	2		1^*^, 3^*^, 5^*^	Control+drought	48.45–56.81	1.49^*^10^−5^	6.77
*QYL.ndsu.1B.1*	4	PH	1^*^, 2^*^, 3^*^, 4	Control+drought	70.08	2.22^*^10^−6^	6.59
*QYL.ndsu.1B.2*	6		1^*^, 5^*^, 6^*^	Control+drought	112.07	3.68^*^10^−5^	6.20
*QYL.ndsu.1D*	7		1, 5^*^	Control+drought	3.40	2.89^*^10^−6^	7.80
*QYL.ndsu.2A*	11	DH	1, 4^*^, 5^*^	Control+drought	144.41	1.86^*^10^−6^	8.08
*QYL.ndsu.2B.1*	13	DH	1, 4^*^, 6^*^	Control+drought	88.93–90.971	1.31^*^10^−5^	5.57
*QYL.ndsu.2B.2*	15	TKW	1, 3, 4, 5, 6	Control+drought	157.21	1.79^*^10^−6^	8.11
*QYL.ndsu.3B*	21	TW, DH	1^*^, 4^*^, 5^*^, 6^*^	Control+drought	62.31–69.53	5.81^*^10^−6^	7.36
*QYL.ndsu.4B*	28	PH, DH, TW	1, 4^*^, 5, 6^*^	Control+drought	56.19	4.17^*^10^−9^	12.04
*QYL.ndsu.5A*	32		1^*^, 4^*^, 6^*^	Control+drought	116.35–117.67	1.7^*^10^−4^	4.11
*QYL.ndsu.5B*	34	PH	1, 4^*^	Control+drought	68.36	1.94^*^10^−6^	6.91
*QYL.ndsu.6A*	36		1, 3^*^, 4^*^	Control+drought	12.48	1.52^*^10^−6^	8.21
*QYL.ndsu.6B.1*	39		1^*^, 4^*^, 5^*^	Control+drought	64.08–64.71	6.08^*^10^−6^	7.33
*QYL.ndsu.6B.2*	40	PH	1, 3^*^, 4	Control+drought	115.25	1.38^*^10^−6^	7.12
*QYL.ndsu.7B*	44	DH	1^*^, 3^*^, 4^*^	Control+drought	89.82–92.52	6.64^*^10^−4^	4.26
*QYL.ndsu.7D*	45		1^*^, 4^*^, 5^*^	Control+drought	128.15–135.55	3.19^*^10^−5^	5.24
*QYL.ndsu.3A*	18	DH	1, 2^*^, 3^*^	Drought	109.95	1.52^*^10^−6^	8.21

*ᵻ DH, Days to heading; PH, Plant height; YLD, Yield; TW, Test weight; TKW, Thousand kernel weight*.

*ǂ1 = Casselton 2012, 2 = Prosper 2012, 3 = Minot 2012, 4 = Prosper 2013, 5 = Prosper 2014, 6 = Hettinger 2014, 7 = Minot 2013, 8 = Mean across environments*.

§ *Position represents the peak point of the QTL interval. The position is based on consensus map of Wang et al. (2014)*.

* *p < 0.001 but above the threshold level*.

Seventeen QTL were identified for YLD. These QTL explained 4.11–12.04% of PV (Table 4). Only one QTL, located on chromosome 4B, had a major effect. Sixteen QTL were identified as constitutive, and the remaining QTL was identified in the drought-prone experimental sites. Five QTL were associated with TW. All of these QTL had minor effects, explaining 3.7–7.66% of PV. All of the QTL identified were constitutive. Seven QTL were identified for TKW, all of which had minor effects, explaining from 5.2 to 9.2% of PV. One QTL among them was constitutive, and the remaining six were identified in the drought-prone environments (Table 4).

The sequence of 69 markers associated with QTL was mined from Wang et al. (2014) to identify their physical position in the Chinese_Spring_IWGSC_RefSeq1.0 (Appels et al., 2018). Out of 69, seven markers (BobWhite_c47948_76, D_contig00840_473, Kukri_c15043_326, Excalibur_c25353_1171, Ex_c3115_2742, Excalibur_rep_c92985_510, Excalibur_c56240_176) were manually anchored on the genome. A total of 47 SNPs were anchored in the predicted gene space, and 22 were found in the intergenic region (Supplementary Table 4). For these intergenic SNPs, we identified the most proximal flanking genes representing the possible linked gene contributing to the phenotype. Thus, a total of 91 AM-QTL-associated most proximal genes along with their predicted function and genome orientation (Table 5) were mined from the genome. Using the expVIP analysis (Ramírez-González et al., 2018, Borrill et al., 2016), we explored the *in silico* expression of these genes affected by drought stress (Supplementary Figure 3) which could be used to prioritize the candidate genes. However, for breeding purposes all the MTAs are valuable.

**Table 5:** List of candidate genes associated with QTL identified in this study.

**QTL and trait**	**Associated/proximal flanking gene**	**Function**	**Gene space?**	**Distal flanking gene**
**Days to heading**				
*QDH.ndsu.1B*	*TraesCS1B01G359800*	Alpha-glucan water dikinase	Yes	
*QDH.ndsu.2A.2*	*TraesCS2A01G421100*	Myosin heavy chain embryonic smooth protein	Yes	
*QDH.ndsu.2B.2*	*TraesCS2B01G395600*	Histone H2A deubiquitinase (DUF3755)	Yes	
*QDH.ndsu.2D*	*TraesCS2D01G076700*	Kinase family protein	Yes	
*QDH.ndsu.3A.1*	*TraesCS3A01G317000*	Vacuolar sorting-associated protein 28-like protein	No	*TraesCS3A01G316900*
*QDH.ndsu.3B*	*TraesCS3B01G356000*	Inositol-1,4,5-trisphosphate 5-phosphatase 4 isoform 1	No	*TraesCS3B01G355900*
*QDH.ndsu.4A.1*	*TraesCS4A01G275500*	Importin-5	Yes	
*QDH.ndsu.4B*	*TraesCS4B01G293600*	Copper-transporting ATPase	Yes	
*QDH.ndsu.4D*	*TraesCS4D01G285000*	50S ribosomal protein L18	Yes	
*QDH.ndsu.5B.2*	*TraesCS5B01G391500*	Evolutionarily conserved C-terminal region 2—RNA binding	Yes	
*QDH.ndsu.6B*	*TraesCS5B01G416700*	Citrate synthase	Yes	
*QDH.ndsu.7B*	*TraesCS7B01G369500*	Tegument protein BRRF2	No	*TraesCS7B01G369400*
*QDH.ndsu.2A.1*	*TraesCS2A01G048900*	Defensin	Yes	
*QDH.ndsu.2A.3*	*TraesCS2A01G517500*	Chloride channel protein	Yes	
*QDH.ndsu.2B.1*	*TraesCS2B01G131500*	THUMP domain-containing protein 1	Yes	
*QDH.ndsu.3A.2*	*TraesCS3A01G425500*	Sucrose-phosphate synthase	Yes	
*QDH.ndsu.4A.2*	*TraesCS4A01G359500*	Squamosa promoter-binding protein	Yes	
*QDH.ndsu.5A.1*	*TraesCS5A01G259200*	Vps52, vacuolar protein sorting-associated protein 52	Yes	
*QDH.ndsu.5A.2*	*TraesCS5D01G381300*	EEIG1/EHBP1 N-terminal domain-containing protein	Yes	
*QDH.ndsu.5B.1*	*TraesCS5B01G021800*	Glutathione s-transferase	Yes	
**Plant height**				
*QPH.ndsu.1A*	*TraesCS1A01G356900*	Choline kinase	Yes	
*QPH.ndsu.1B*	*TraesCS1B01G309400*	Non-specific serine/threonine protein kinase	Yes	
*QPH.ndsu.2A.1*	*TraesCS2A01G247000*	D-Lactate dehydrogenase	No	*TraesCS2A01G246900*
*QPH.ndsu.2A.2*	*TraesCS2A01G559200*	1-Phosphatidylinositol-3-phosphate 5-kinase	Yes	
*QPH.ndsu.2B*	*TraesCS2A01G452200*	Ribosome biogenesis protein BRX1-like protein	No	*TraesCS2A01G452300*
*QPH.ndsu.3A.1*	*TraesCS3A01G087800*	Transcription factor GTE9	Yes	
*QPH.ndsu.3A.2*	*TraesCS3B01G491000*	F-box protein	Yes	
*QPH.ndsu.3A.3*	*TraesCS3A01G525300*	P-loop-containing nucleoside triphosphate hydrolase superfamily	No	*TraesCS3A01G525200*
*QPH.ndsu.3B*	*TraesCS3B01G531400*	Voltage-dependent L-type calcium channel subunit	Yes	
*QPH.ndsu.3D.1*	*TraesCS3B01G004600*	Subtilisin-like protease	Yes	
*QPH.ndsu.3D.2*	*TraesCS3D01G250900*	ATP synthase subunit beta	Yes	
*QPH.ndsu.4B*	*TraesCS4B01G047900*	NADH-ubiquinone oxidoreductase subunit	Yes	
*QPH.ndsu.5B*	*TraesCS5B01G303100*	Hydroxyacylglutathione hydrolase	Yes	
*QPH.ndsu.6A.2*	*TraesCS6A01G398000*	Protein KINESIN LIGHT CHAIN-RELATED 3	Yes	
*QPH.ndsu.6B.1*	*TraesCS6B01G126900*	Kinetochore protein nuf2	Yes	
*QPH.ndsu.7A.2*	*TraesCS7A01G540200*	Receptor-like protein kinase	Yes	
*QPH.ndsu.6A.1*	*TraesCS6A01G297400*	B-cell receptor-associated like protein	No	*TraesCS6A01G297500*
*QPH.ndsu.6B.2*	*TraesCS6B01G434900*	ABC transporter G family member	Yes	
*QPH.ndsu.6D*	*TraesCS6D01G013700*	Zinc finger BED domain-containing protein DAYSLEEPER	Yes	
*QPH.ndsu.7A.1*	*TraesCS6A01G073600*	DNA topoisomerase 3-alpha	No	*TraesCS6A01G073500*
**1K kernel weight**				
*QTKW.ndsu.2B.1*	*TraesCS2B01G491600*	Transmembrane protein	Yes	
*QTKW.ndsu.2B.2*	*TraesCS2B01G606100*	Receptor kinase 1	No	*TraesCS2B01G606200*
*QTKW.ndsu.4A.1*	*TraesCS4A01G269200*	TBPIP	No	*TraesCS4A01G269400*
*QTKW.ndsu.4A.2*	*TraesCS4A01G365500*	Protein PLANT CADMIUM RESISTANCE 2	Yes	
*QTKW.ndsu.4A.3*	*TraesCSU01G167400*	Receptor kinase	No	*TraesCSU01G167500*
*QTKW.ndsu.6B*	*TraesCS6B01G228100*	RNA-binding protein	No	*TraesCS6B01G228300*
*QTKW.ndsu.5B*	*TraesCS5B01G015100*	WAT1-related protein	No	*TraesCS5B01G015200*
**Test weight**				
*QTW.ndsu.1A*	*TraesCS1D01G020000*	Actin	No	TraesCS1D01G020100
*QTW.ndsu.2A*	*TraesCS2A01G012200*	Protein transport Sec24-like	Yes	
*QTW.ndsu.3A*	*TraesCS3A01G149800*	Dual-specificity protein phosphatase	No	*TraesCS3A01G149900*
*QTW.ndsu.3B*	*TraesCS3B01G153500*	Arginine-tRNA ligase	No	*TraesCS3B01G153600*
*QTW.ndsu.4B*	*TraesCS4D01G050100*	26S proteasome non-ATPase regulatory subunit	No	*TraesCS4D01G050200*
**Yield**				
*QYL.ndsu.1A*	*TraesCS1A01G040600*	Glucose-6-phosphate isomerase	Yes	
*QYL.ndsu.1B.1*	*TraesCS1B01G252700*	RCC1 family with FYVE zinc finger domain-containing protein	Yes	
*QYL.ndsu.1B.2*	*TraesCS1B01G253500*	UDP-sulfoquinovose synthase	Yes	
*QYL.ndsu.1D*	*TraesCS1A01G002300*	Endoribonuclease E-like protein	Yes	
*QYL.ndsu.2A*	*TraesCS2D01G541500*	Regulatory protein recX	Yes	
*QYL.ndsu.2B.1*	*TraesCS2D01G126100*	Cellulose synthase	Yes	
*QYL.ndsu.2B.2*	*TraesCS2A01G567900*	Tetratricopeptide repeat	Yes	
*QYL.ndsu.3B*	*TraesCS3B01G153500*	Arginine-tRNA ligase	No	*TraesCS3B01G153600*
*QYL.ndsu.4B*	*TraesCS4B01G047900*	NADH-ubiquinone oxidoreductase subunit	Yes	
*QYL.ndsu.5A*	*TraesCS7B01G206200*	Calcium-binding family protein	No	*TraesCS7B01G206300*
*QYL.ndsu.5B*	*TraesCS5D01G326500*	Alpha/beta hydrolase	Yes	
*QYL.ndsu.6A*	*TraesCS6D01G011300*	DNA-directed RNA polymerase subunit beta	No	*TraesCS6D01G011400*
*QYL.ndsu.6B.1*	*TraesCS6B01G173000*	Sucrose-phosphate synthase	Yes	
*QYL.ndsu.6B.2*	*TraesCS6B01G455400*	Tuftelin-interacting protein 11	No	*TraesCS6B01G455500*
*QYL.ndsu.7B*	*TraesCS7B01G363600*	Geranylgeranyl transferase type-2 subunit alpha 1	Yes	
*QYL.ndsu.7D*	*TraesCS7D01G287700*	Copia-like polyprotein/retrotransposon	No	*TraesCS7D01G287800*
*QYL.ndsu.3A*	*TraesCS3A01G406700*	P-loop containing nucleoside triphosphate hydrolases superfamily protein	Yes	

## Discussion

### Association Analyses

Studies are conducted to dissect the genetics of drought tolerance in many crops including wheat, and it is well-known that drought tolerance is a complex quantitative trait affected by genetic and environmental factors (Gahlaut et al., 2019). In this study, the iBLUP method (Yang et al., 2014b) was used to impute missing genotypic data as it was reported to tolerate a high rate of missing data especially for rare alleles, compared to the common imputation methods. High-density single-nucleotide polymorphism (SNP) genotyping arrays explore genomic diversity and MTAs very efficiently (Wang et al., 2014). Infinium iSelect 90K assay uses more than 81,000 gene-associated SNPs to reveal polymorphism in allohexaploid wheat populations (Wang et al., 2014; Kumar et al., 2016, 2019). Higher genome coverage and resolution in the dissection of wheat's agronomic traits are possible using this genotypic tool (Kirigwi et al., 2007; Alexander et al., 2012; Ibrahim et al., 2012b). The marker density found in this study (0.49 cM/marker) was in agreement with the previous studies using the 90K Infinium iSelect assay (Wang et al., 2014; Ain et al., 2015; Kumar et al., 2016). The MLM model used in this association study has been proven to be very efficient for genome-wide association studies (GWAS) and can be used with either structure (R) or principal component (PC) analyses. This study used five PCs, which captured 25% of the variation. The MLM model, which accounts for both structure and relatedness (PC + K), was used for the marker–trait association study.

Determining the threshold for the *p*-value is crucial. A liberal threshold will declare a false-positive association (a type I error), whereas a too stringent threshold is likely to miss a true association (a type II error). Taking this into consideration, the initial cutoff was chosen as *p* ≤ 0.001, which was not very stringent. Then, the threshold (*p* ≤ 3.4 ^*^ 10^−6^) was determined using the Bonferroni correction (Yang et al., 2014a), which was very stringent. The MTAs identified at the initial cutoff and the threshold were reported if they were identified in at least two environments. This repetition of the MTA further minimized any false associations.

### Use of Secondary Data to Assess Drought Conditions

Drought can be assessed by variable weather conditions, soil moisture, and crop conditions over a particular growing season (Lanceras et al., 2004). Therefore, rainfall data were collected, and the soil types of the experimental sites, which reflect soil moisture, were taken into consideration to assess drought conditions for this study. The total amount of rainfall was collected from planting date to plant physiological maturity. The dates for the physiological maturity of the plants were calculated by adding 30 days to DH (Simmons et al., 1914). Among the experimental locations, Casselton 2012, Prosper 2012, and Minot 2012 were considered to have drought conditions, whereas Prosper 2013, Minot 2013, Prosper 2014, and Hettinger 2014 were considered to have control or normal conditions. Although Minot 2012 and Prosper 2014 had about the same amount of rainfall, the soil in Prosper had a better water-holding capacity. Therefore, Minot 2012 was considered to have drought conditions.

### Use of Agronomic Data to Assess Drought Tolerance

#### Days to Heading

Several major and minor QTL were revealed for DH, which indicated the quantitative nature of the trait. The eight QTL for DH, identified exclusively under drought conditions, could play a vital role in drought tolerance. Also, the constitutive QTL can be used for drought tolerance breeding in wheat. Some of these QTL (exclusively expressed under drought conditions, called constitutive QTL) identified in this study likely correspond with some already reported QTL associated with drought tolerance; however, further studies such as allelism test is warranted to determine their relationship. Malik et al. (2015) identified three adjacent QTL on chromosome 2A for drought tolerance related to the photosynthetic rate, cell membrane stability, and relative water content. The QTL *QDH.ndsu.2A.1* in this study likely represent one of those QTL previously reported (Malik et al., 2015; Gahlaut et al., 2019), but further studies are required to determine their relationship. Two QTL identified in this study on the chromosome 3A which were important for drought tolerance, *QDH.ndsu.3A.1* and *QDH.ndsu.3A.2*, could represent the QTL *QHea.T84-3A* which was earlier found to increase DH under both drought and non-drought conditions (Ibrahim et al., 2012b). Chromosomal arm 3AL also harbors a gene for earliness *per se* (Edae et al., 2014), associated with enhanced response to abscisic acid (*ERA1*), which provides drought tolerance (Edae et al., 2014). The gene *ERA1*, also located on chromosome 3B, could represent the QTL *QDH.ndsu.3B* identified in this study which is closely associated with *TraesCS3B01G356000* (Table 5), putatively encoding for inositol-1,4,5-trisphosphate 5-phosphatase (InsP_3_). InsP_3_ is reported to be a second messenger in plants responding to many stimuli and has been shown to affect drought tolerance, carbohydrate metabolism, and phosphate-sensitive biomass increases in tomato (Khodakovskaya et al., 2010). In wheat, differential expression of the *phospholipase C* gene regulating the inositol-1,4,5-triphosphate (IP_3_) signal transduction pathway possibly results in the quick sensing of drought stress (Ergen et al., 2009). Kamran et al. (2013) identified a QTL, *QFlt.dms-4A.1*, for reduced DH at 61.2 cM on chromosome 4A, which may represent the constitutive QTL *QDH.ndsu.4A.1* identified in this study. The constitutive QTL *QDH.ndsu.2B.2* is located in the same region as the earlier reported QTL *QCrs-* (Ibrahim et al., 2012a), which deteriorate the number of root crossing in both water regimes. A QTL for drought tolerance on 4AL reported earlier by Alexander et al. (2012) may represent the QTL *QDH.ndsu.4A.2*, which was identified exclusively for drought-prone environments in this study. The constitutive QTL *QDH.ndsu.6B* was located in the same genomic location as the QTL *QHea*+, which was reported to reduce DH under both water conditions (Ibrahim et al., 2012b). Huang et al. (2006) reported a QTL for days to maturity, *QDtm.crc-2D*, which corresponded with the constitutive QTL in this study, *QDH.ndsu.2D*, representing the kinase family protein. However, the SNP markers associated with sucrose-phosphate synthase (*TraesCS3A01G425500*) and vacuolar protein sorting-associated protein (*TraesCS5A01G259200, TraesCS3A01G317000*) in this group (Table 5) may represent the important abiotic stress genes controlling the plant height. The QTL *QDH.ndsu.5B.2* and *QDH.ndsu.7B* identified in this study seem to be novel.

#### Plant Height

The QTL *QPH.ndsu.5B* could represent the ortholog to the GA-insensitive dwarf gene, *GID1L2*, in rice, indicating the synergistic relationship of rice and wheat (Zanke et al., 2014). The major QTL for PH, *QPH.ndsu.6B.1* and *QPH.ndsu.6B.2*, have been identified on wheat chromosome 6B, and several previous studies also reported QTL for PH on a similar location (Zanke et al., 2014; Gahlaut et al., 2019; Abou-Elwafa and Shehzad, 2021). The major QTL *QPH.ndsu.4B* could represent the reduced height gene *Rht-B1* (Wilhelm, 2011), which was reported to be on the short arm of chromosome 4B. This gene encodes the DELLA protein that reduces a plant's sensitivity to gibberellin (GA), thereby reducing stalk length and making the plant semi-dwarf. The QTL *QPH.ndsu.1A, QPH.ndsu.2A.1, QPH.ndsu.6A.2*, and *QPH.ndsu.3A.3* could represent the QTL for PH reported earlier by Zanke et al. (2014). The QTL *QPH.ndsu.3A.2* and *QPH.ndsu.3D.2*, important for drought tolerance, could be the same as those reported by Ibrahim et al. (2012a). Liu et al. (2011) identified a QTL for PH, *QHt-3B*, which could occupy the same region as the QTL *QPH.ndsu.3B* in the study. The QTL *QPH.ndsu.7A.1* coincided with the earlier reported QTL *QHt.crc-7A* (McCartney et al., 2005). The QTL *QPH.ndsu.7A.2* in a receptor-like kinase gene (*TraesCS7A01G540200*) and *QPH.ndsu.3D.1* in a subtilisin-like protease (Table 5) in this study did not correspond with any reported QTL and hence could be novel.

#### YLD

In the past, Edae et al. (2014) reported a QTL for TKW on chromosome 1BL and a QTL for TW on chromosome 2BL that could be in the region of QTL *QYL.ndsu.1B.1* and *QYL.ndsu.2B.2*, respectively. More recently, Tura et al. (2020) detected a main-effect QTL, *QYld.aww-1B.2*, on 1B chromosome which likely represents the same locus as *QYL.ndsu.1B*.1; however, further research is warranted to determine their relationship. Ibrahim et al. (2012a) identified a QTL, *QCrs.D84-2B*, on chromosome 2B at 93.4 cM that deteriorates the number of root crossings under both water regimes and could represent the QTL *QYL.ndsu.2B.1* (*TraesCS2D01G126100*-cellulose synthase) found in this study. Another QTL for YLD *QYld.T84-3Bat* identified earlier (Ibrahim et al., 2012b) occupies the same location as the QTL *QYL.ndsu.3B* identified in this study. Another QTL, *QYld.T84-3Bat* 59.8, which deteriorated YLD under both water regimes, could coincide with the QTL *QYL.ndsu.4B* in (*TraesCS4B01G047900*) identified in this study. The QTL *QYL.ndsu.5B* and *QYL.ndsu.6B.2* also likely correspond to the QTL for TW and TKW identified earlier by Edae et al. (2014). Also, the QTL *QYL.ndsu.5B* corresponded with the QTL QYld^*^, which was reported to improve YLD under drought stress (Ibrahim et al., 2012b). The QTL *QYL.ndsu.1B.2* had the same genomic location as the constitutive QTL for the green leaf area reported by Edae et al. (2014). Ibrahim et al. (2012a) reported a QTL, *QTgw*+, which improved thousand-grain weight under both water conditions and could represent the QTL *QYL.ndsu.1D*. The QTL *QYL.ndsu.2A* likely coincides with the YLD QTL *QGY.caas-2A* (Li et al., 2015) or a QTL identified by Mathew et al. (2019) evaluated under drought stress conditions. Huang et al. (2006) identified the QTL *QTgw.crc-6A* for TKW that seems to be present at the same location as the QTL *QYL.ndsu.6A* identified in this study. The QTL *QYL.ndsu.7D* corresponded with the QTL *QHi*+, which was reported to improve the harvest index under both water conditions (Ibrahim et al., 2012b).

#### TW

The QTL *QTW.ndsu.4B* could be the same QTL earlier reported by Li et al. (2016) on the same chromosome. The QTL *QTW.ndsu.1A* corresponded with two QTL for YLD, *QYld.abrii-1A1.2* (Azadi et al., 2014) and *QGY.caas-1A* (Li et al., 2015). The constitutive QTL *QTW.ndsu.2A* occupied the same genomic region as the QTL for drought tolerance related to the photosynthetic rate reported by Malik et al. (2015). The QTL *QTW.ndsu.3B* corresponded with the YLD QTL *QYld.T84-3Bat* reported by Ibrahim et al. (2012b).

#### TKW

The QTL *QTKW.ndsu.4A.2* had the same genomic location as the QTL reported by Kirigwi et al. (2007) for YLD and YLD-related traits under drought stress. Ibrahim et al. (2012a) identified the *QTgw-* for thousand-grain weight under both water conditions, which seems to represent the QTL *QTKW.ndsu.6B* identified in this study. The QTL *QTKW.ndsu.2B.1, QTKW.ndsu.2B*.2, and *QTKW.ndsu.4A.3* could be the same QTL for thousand-grain weight reported by Zanke et al. (2015). The QTL *QTKW.ndsu.4A.1* and *QTKW.ndsu.5B* seem to be novel QTL as they do not correspond with any reported QTL.

## Conclusions

This study revealed 69 QTL, which included 50 constitutive QTL, three QTL identified for the control water regime, and 16 QTL exclusively under the drought conditions (Table 4; Supplementary Figure 2). These 16 QTL could be used for developing lines suitable for drought conditions. We also reported the QTL-associated genes, their physical positions, and predicted functions along with *in silico* expression prediction in abiotic stress conditions (Supplementary Figure 3). Chromosome 5B, 6B, and 4B seem to be very important for drought tolerance by reducing PH and increasing YLD and YLD-related traits. Several identified QTL occupied genomic regions reported earlier for earliness *per se*, drought tolerance, and reduced height. The consistency of some QTL in the different environments indicated their validity. Overall, this study provides valuable genetic and genomic resources to the breeders to design programs to breed drought-tolerant wheat cultivars, combining traditional and genomics-based approaches.

## Data Availability Statement

The list of markers and their genotypic data is available at figshare, doi: 10.6084/m9.figshare.14195348 and also in the Supplementary Table 3.

## Author Contributions

SR: data collection, analysis, and major write-up of the manuscript. SM: data collection and analysis. AK: data analyses, manuscript write-up, and review. EE, SK, and SSi: data analysis and manuscript review. MA and AM: data and manuscript review. SSo and SSa: data analysis and write-up. MM: conceptualization and design of the experiment, phenotypic data collection, and manuscript write-up and review. All authors contributed to the article and approved the submitted version.

## Conflict of Interest

The authors declare that the research was conducted in the absence of any commercial or financial relationships that could be construed as a potential conflict of interest.
