# Supplementary material for: Genome-Wide Association Mapping for Yield and Related Traits Under Drought Stressed and Non-stressed Environments in Wheat
Source: Front Genet. 2021 Jun 22;12:649988. doi: 10.3389/fgene.2021.649988 (PMC8258415; doi:10.3389/fgene.2021.649988)
Supplement: Supplementary file 2 [file Data_Sheet_2.docx]

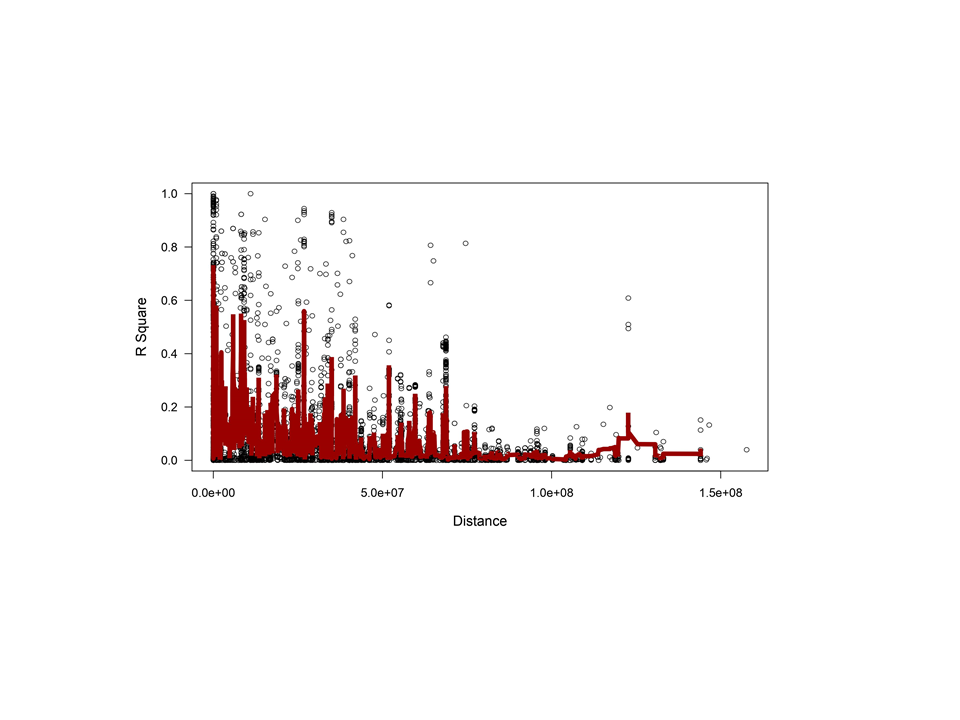


**Figure S1.** Linkage disequilibrium (LD) measured by calculating r^2^ against physical distance (in bp) between pairs of single-nucleotide polymorphic (SNP) marker in a panel of 361 hard red spring wheat genotypes.


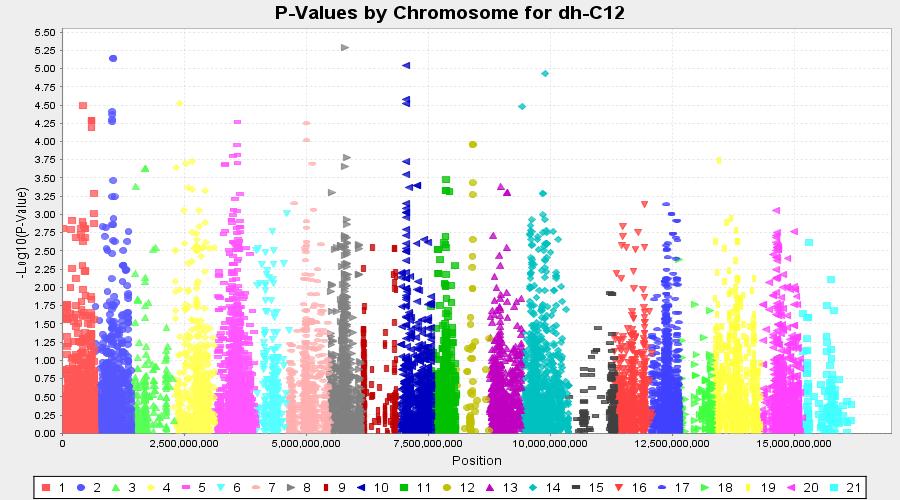


DH-Casselton, 2012


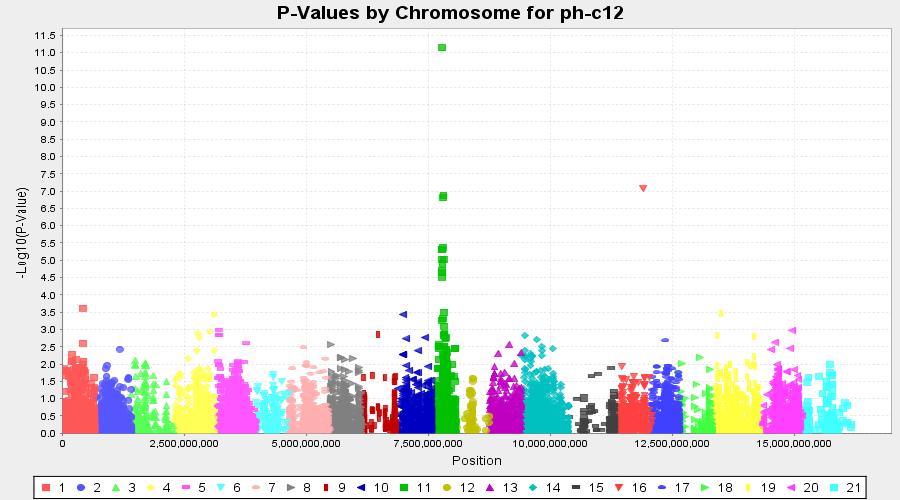


PH-Casselton, 2012


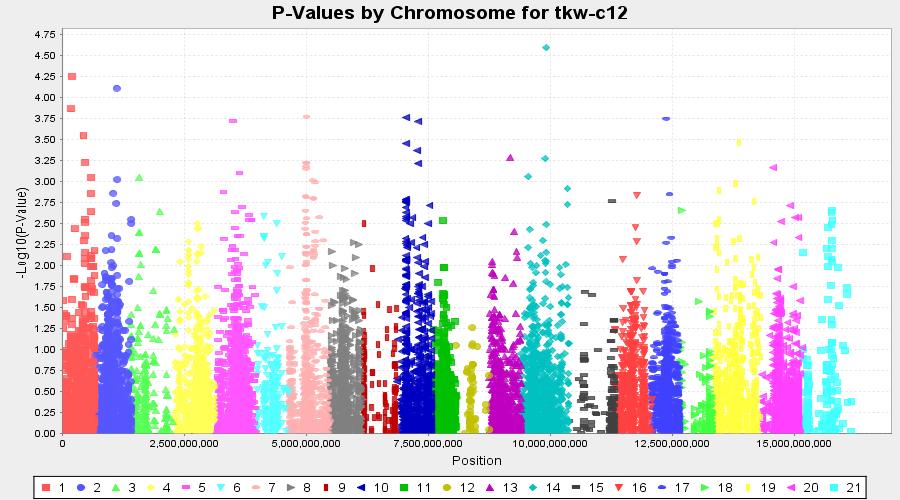


TKW-Casselton, 2012


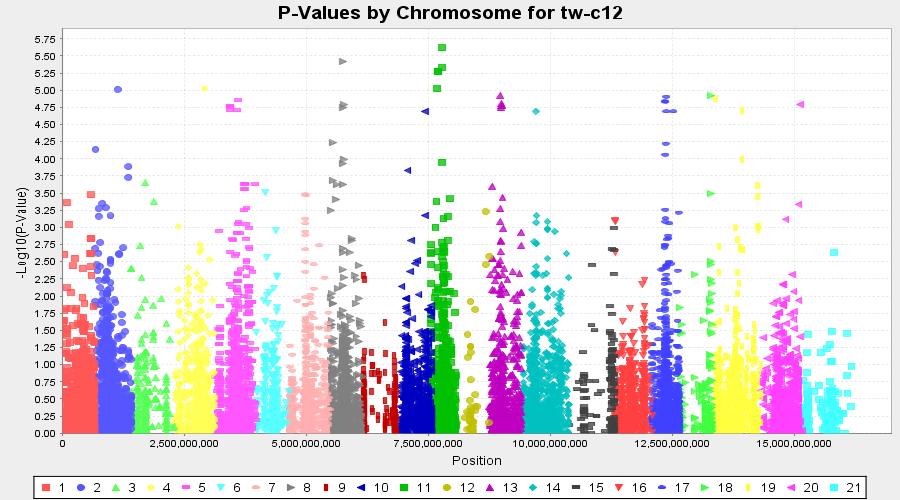


TW-Casselton, 2012


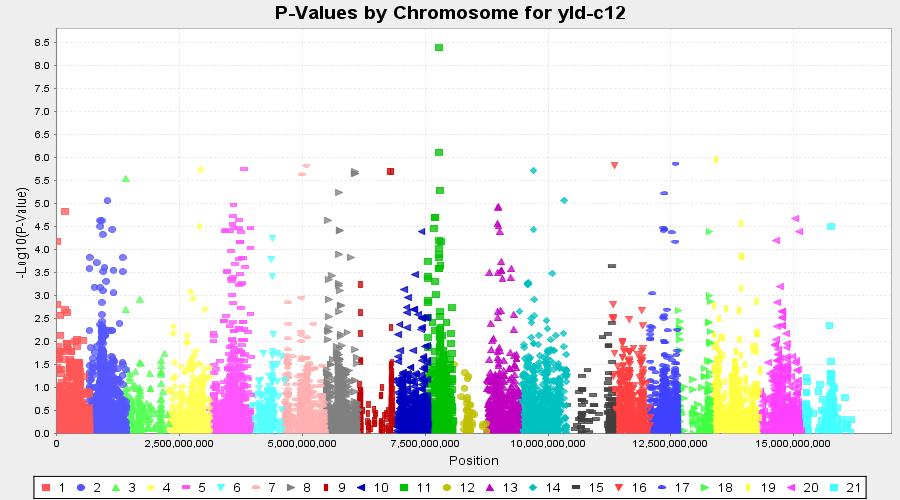


YLD-Casselton, 2012


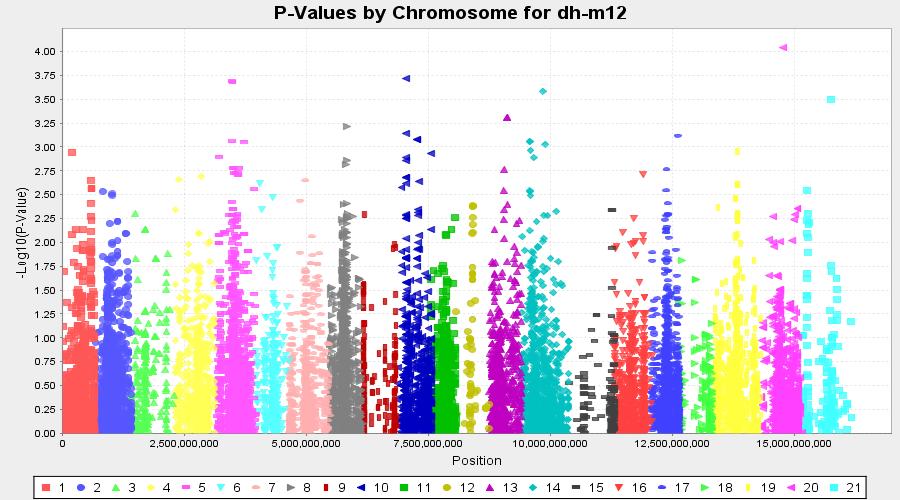


DH-Minot, 2012


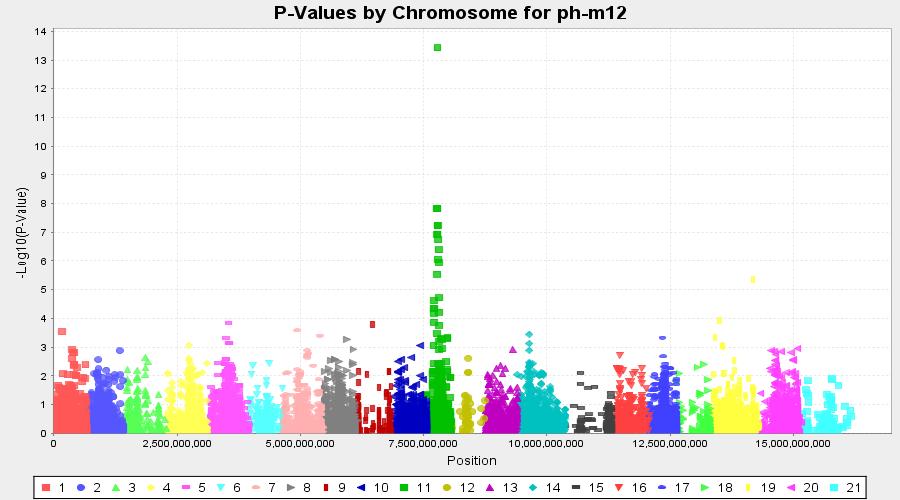


PH-Minot, 2012


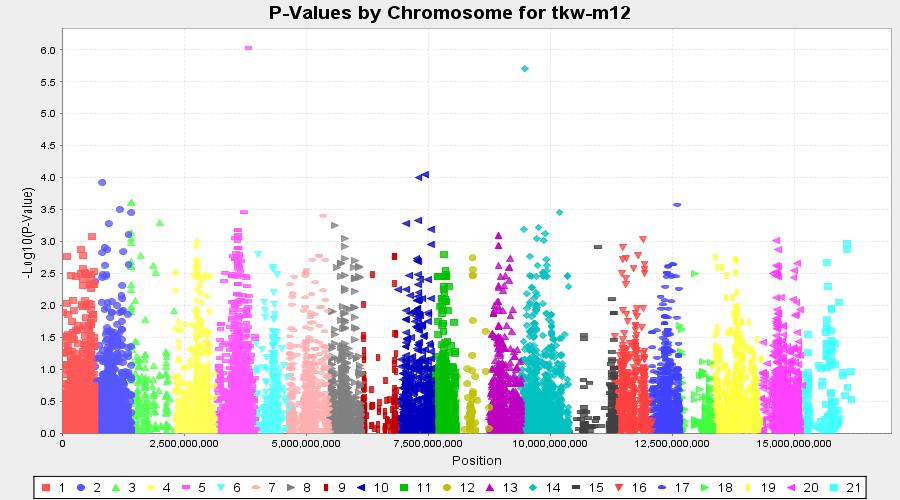


TKW-Minot, 2012


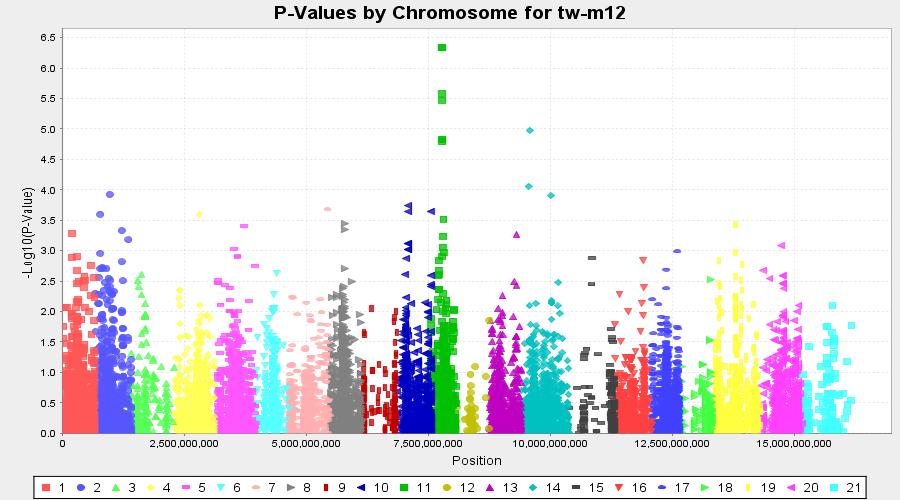


TW-Minot, 2012


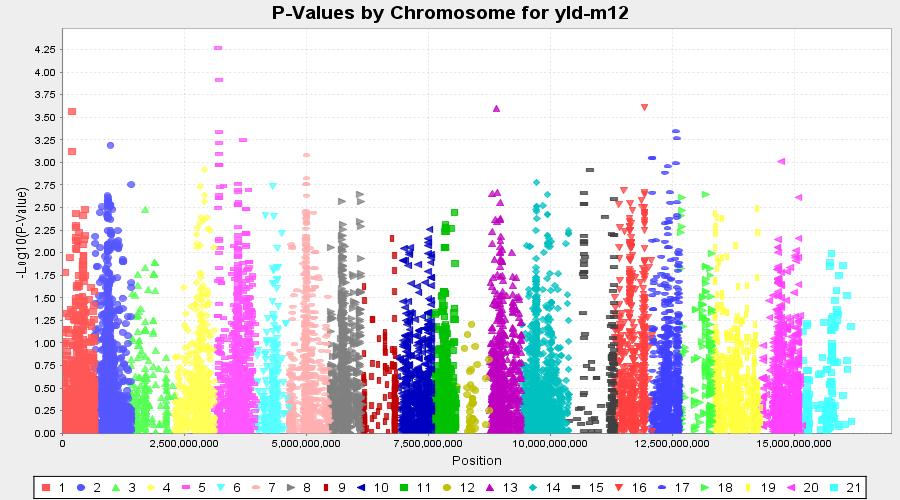


YLD-Minot, 2012


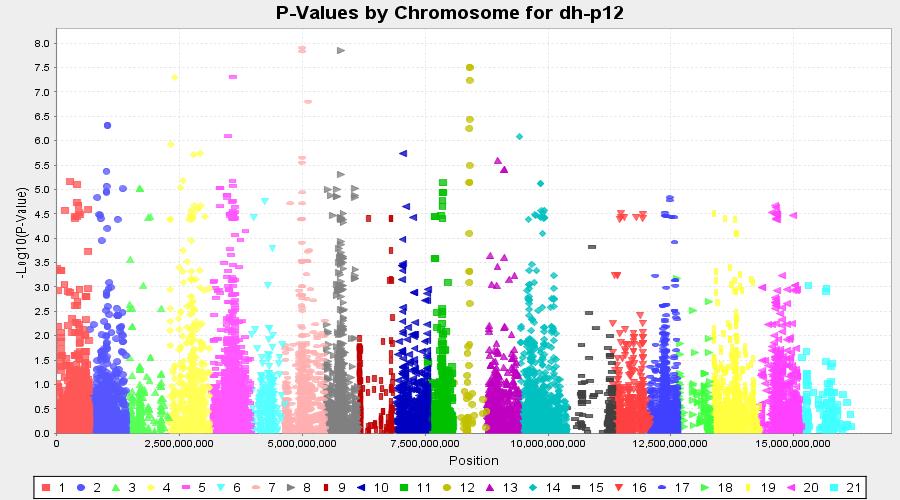


DH-Prosper, 2012


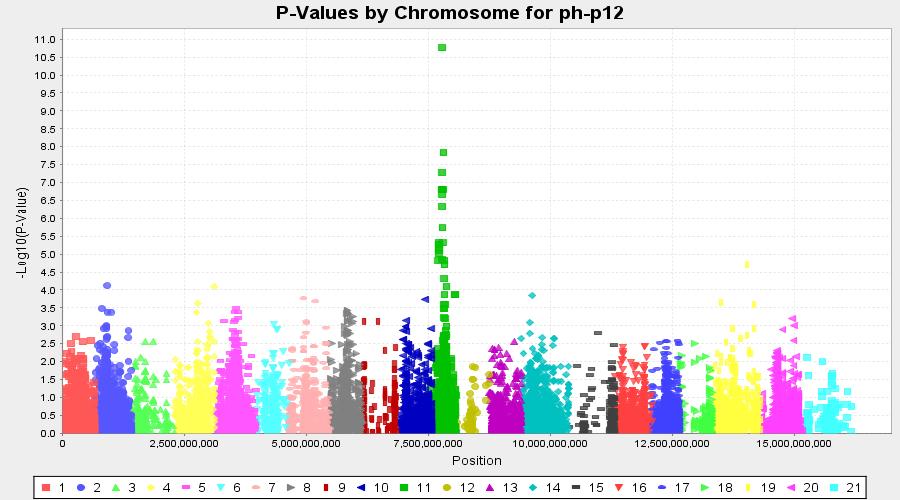


PH-Prosper, 2012


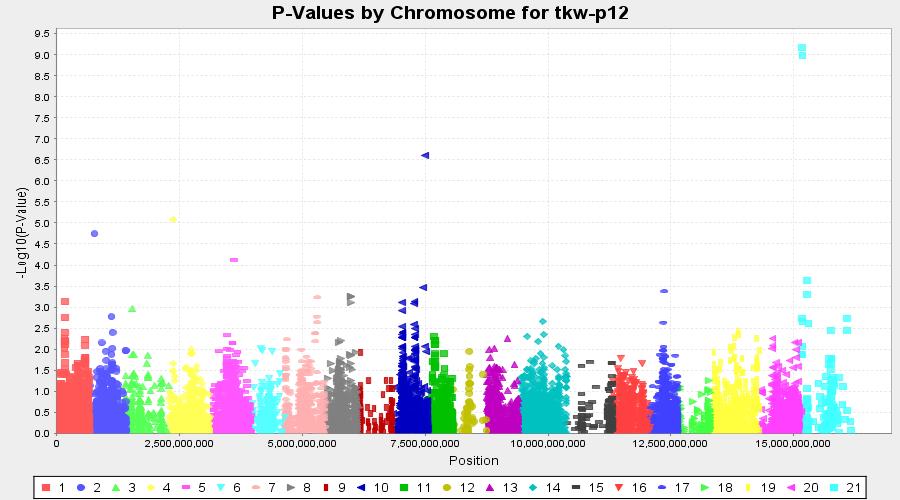


TKW-Prosper, 2012


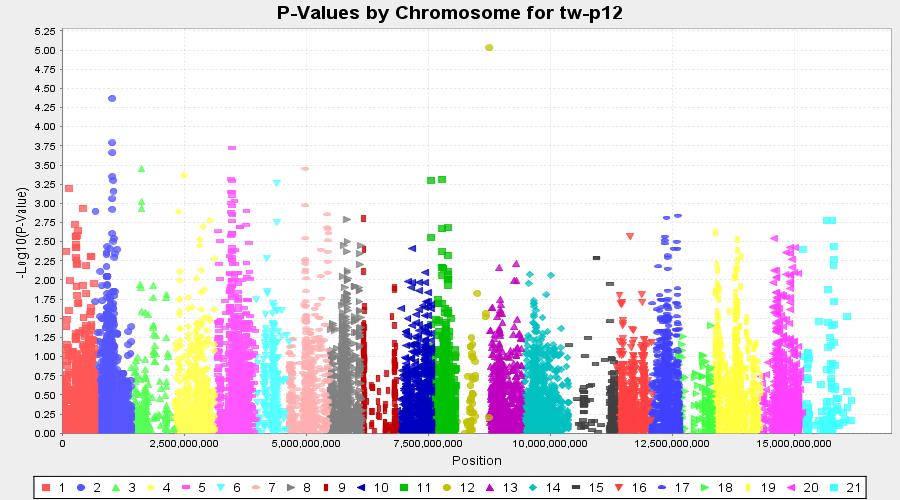


TW-Prosper, 2012


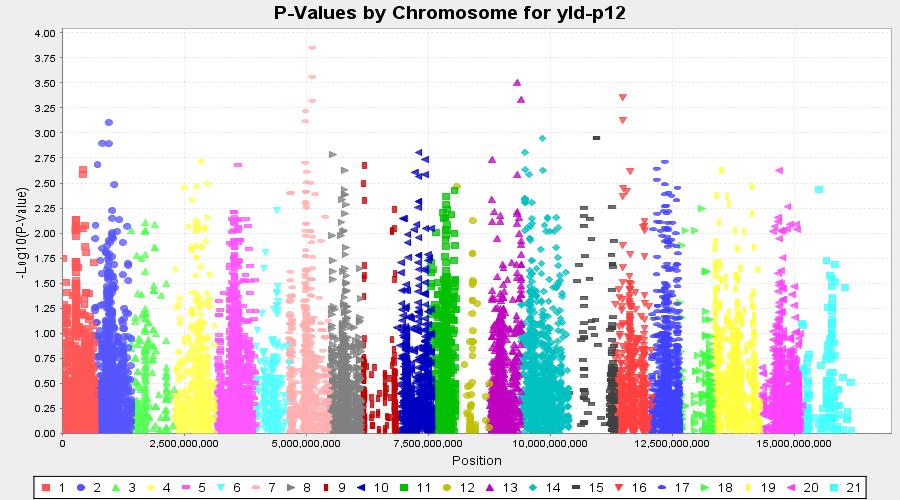


YLD-Prosper, 2012


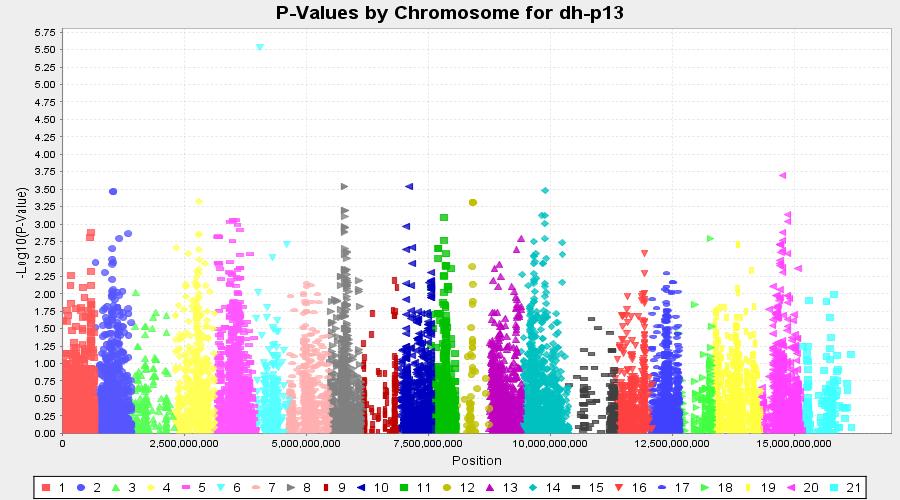


DH-Prosper, 2013


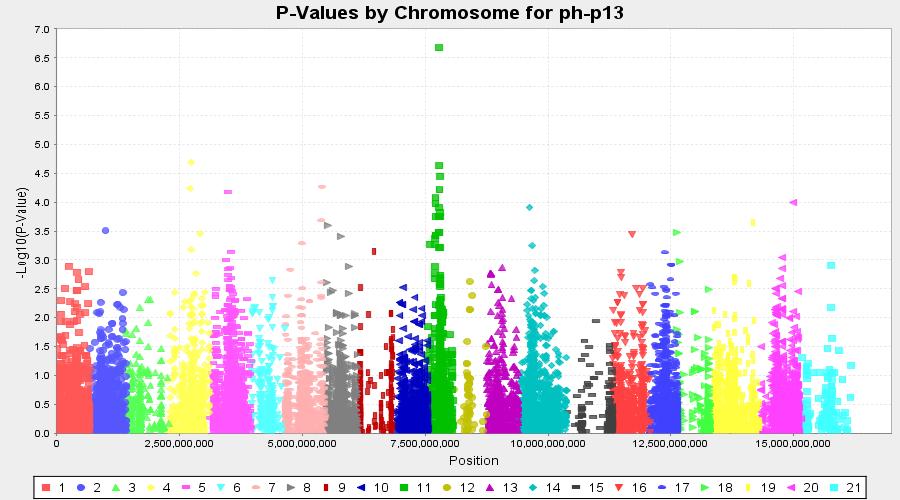


PH-Prosper, 2013


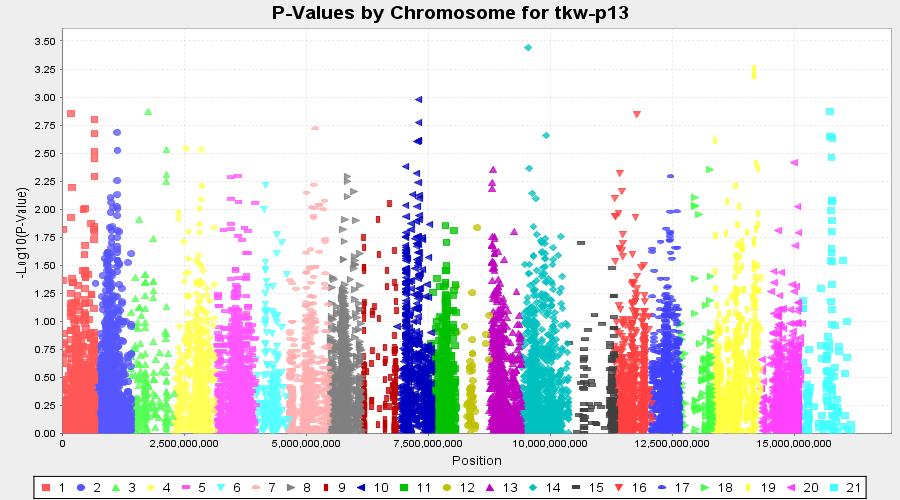


TKW-Prosper, 2013


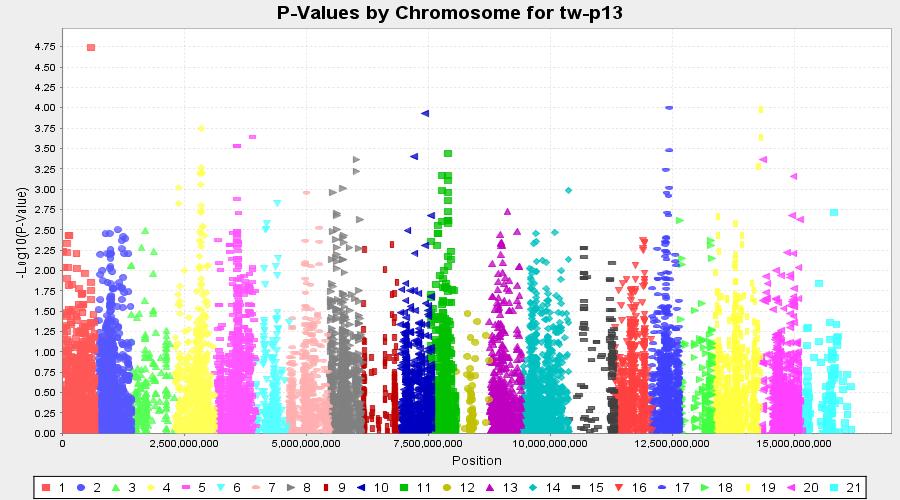


TW-Prosper, 2013


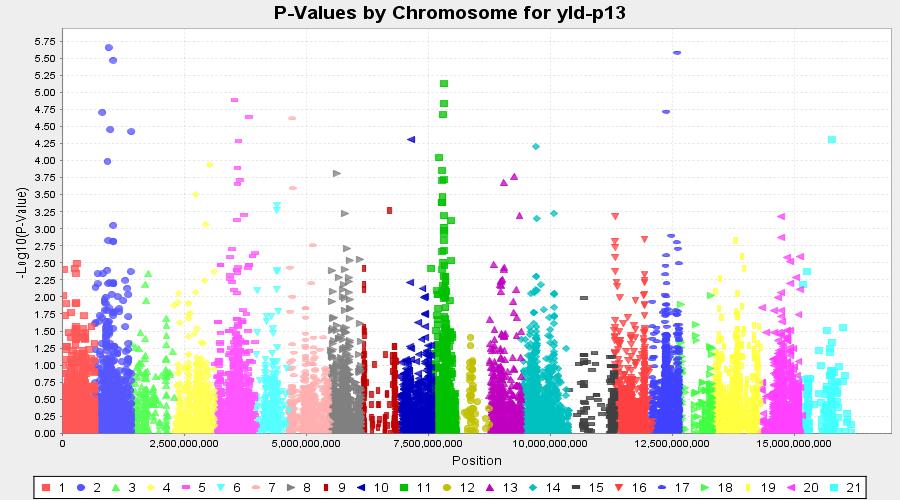


YLD-Prosper, 2013


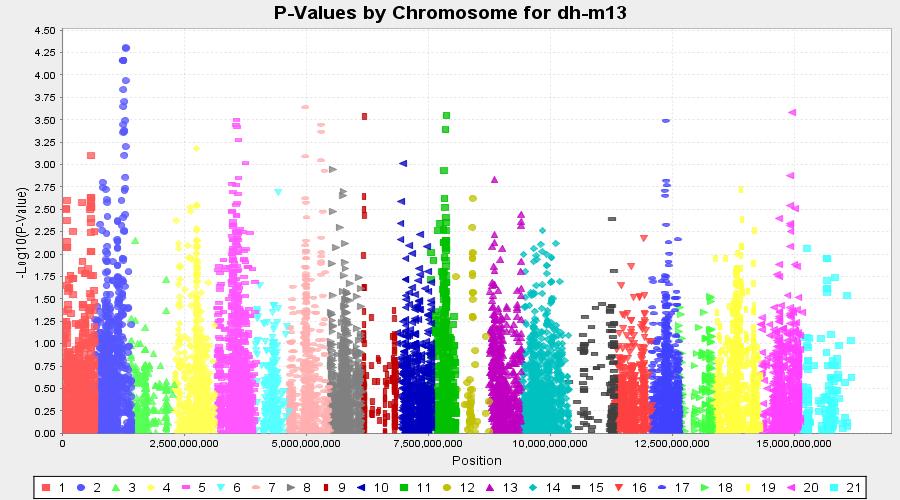


DH-Minot, 2013


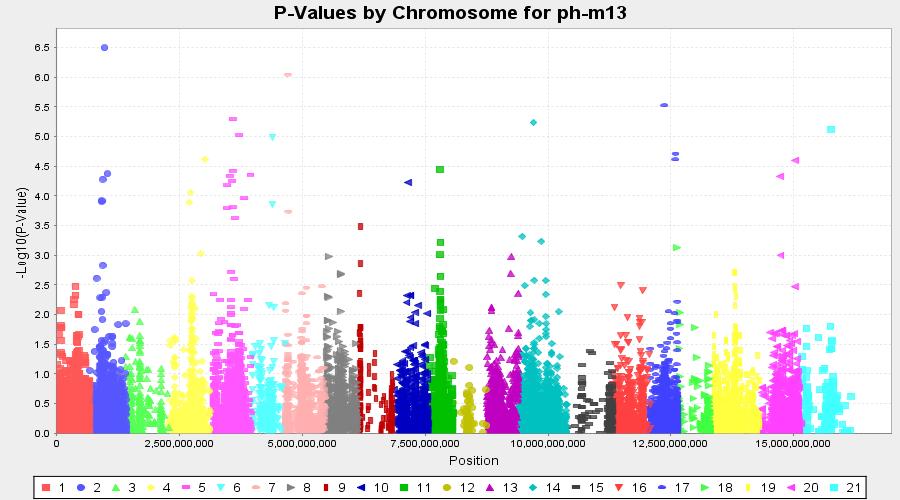


PH-Minot, 2013


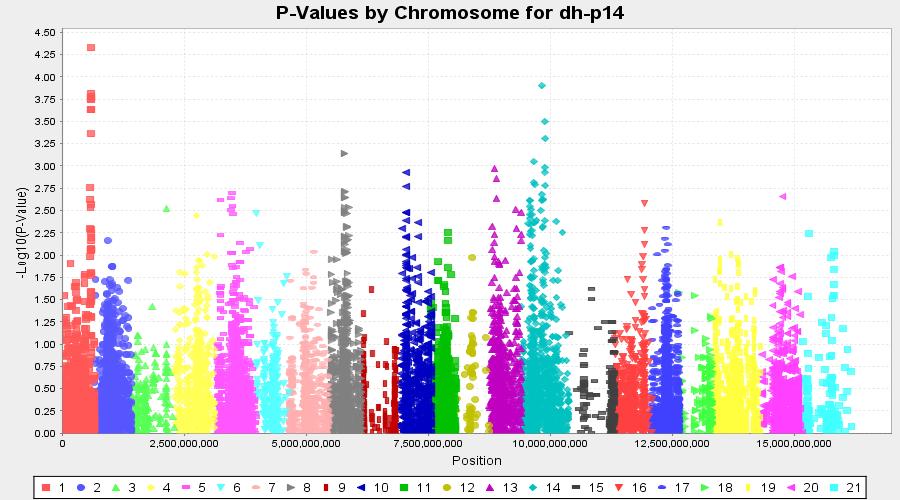


DH-Prosper, 2014


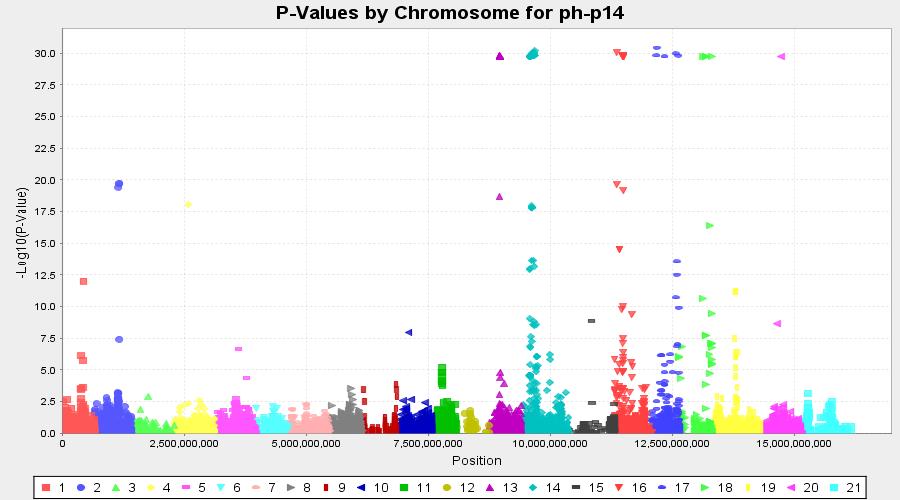


PH-Prosper, 2014


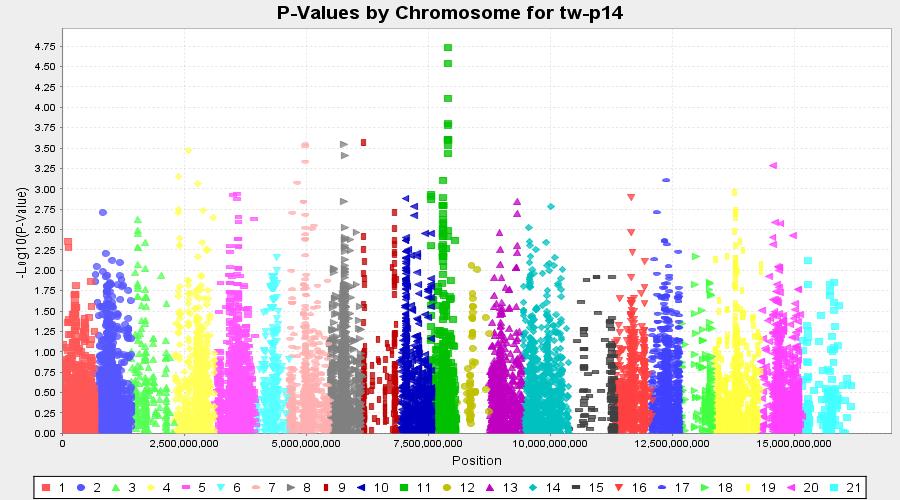


TW-Prosper, 2014


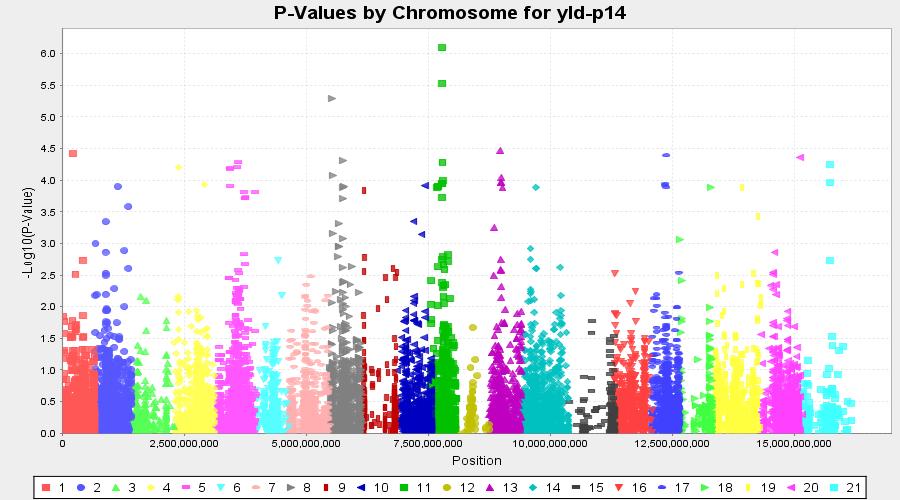


YLD-Prosper, 2014


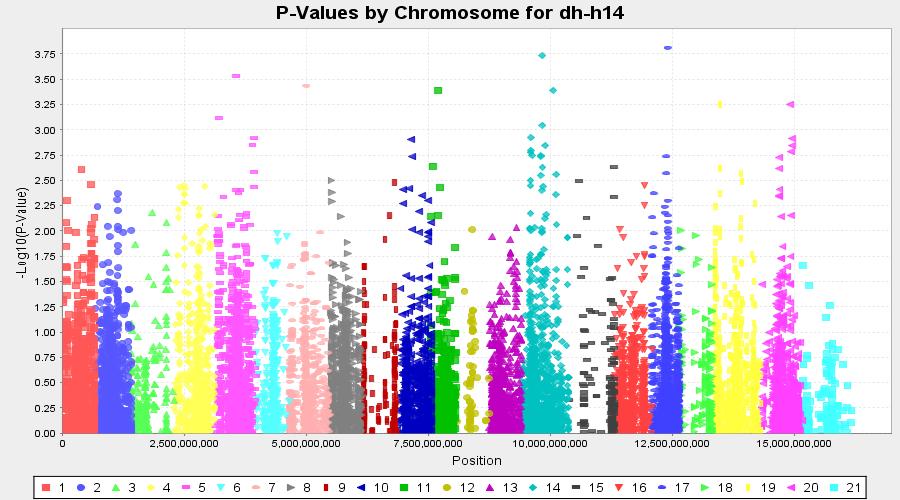


DH-Hettinger, 2014


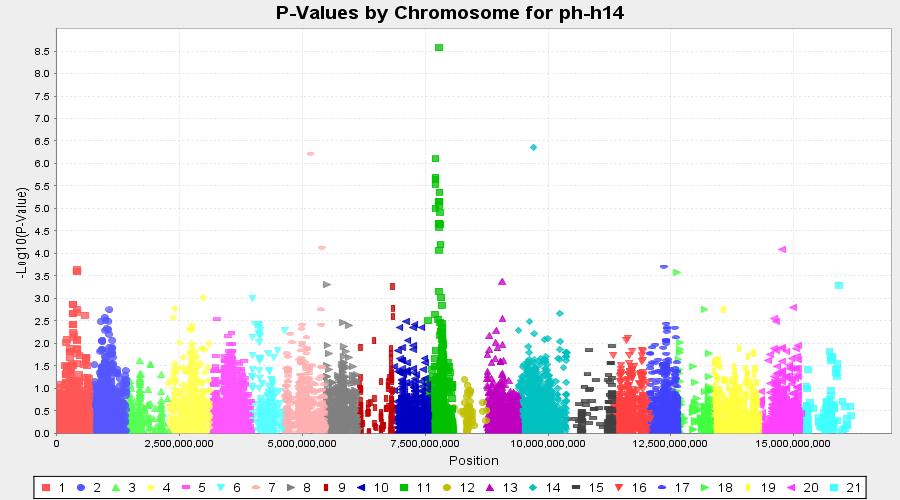


PH-Hettinger, 2014


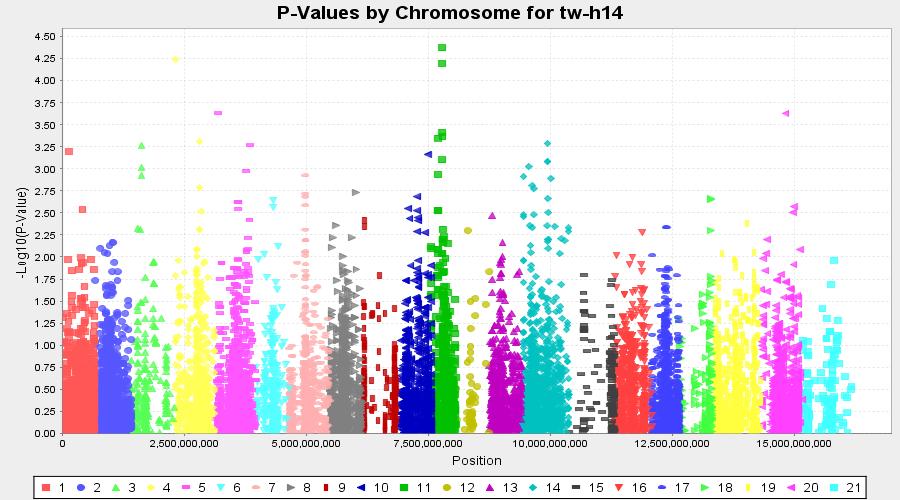


TW-Hettinger, 2014


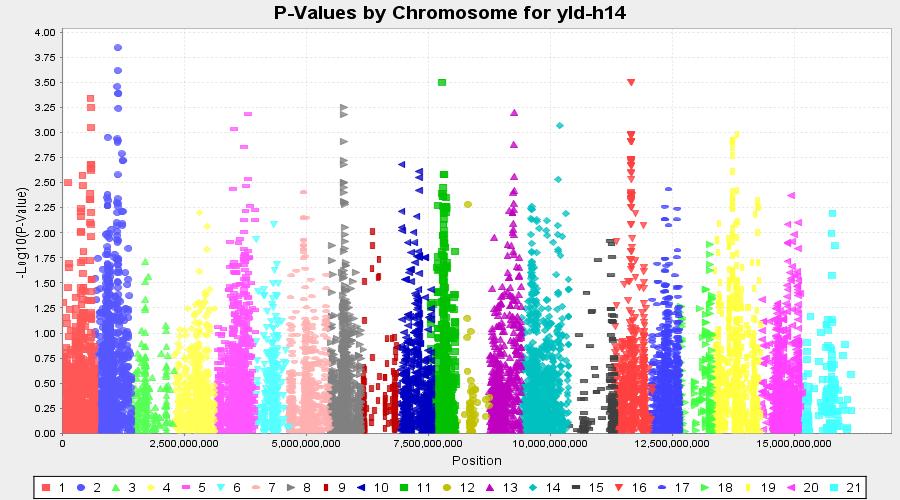


YLD-Hettinger, 2014

# Figure S2. Manhattan plot demonstrating genome-wide SNP loci associated with yield and yield related traits in hard red spring wheat panel. The panel was evaluated for yield and yield related traits in 9 different location of North Dakota in 2012, 2013, and 2014. PH, plant height; DH, days to heading; YLD, yield; TW, test weight; and TKW, thousand kernel weight.

**
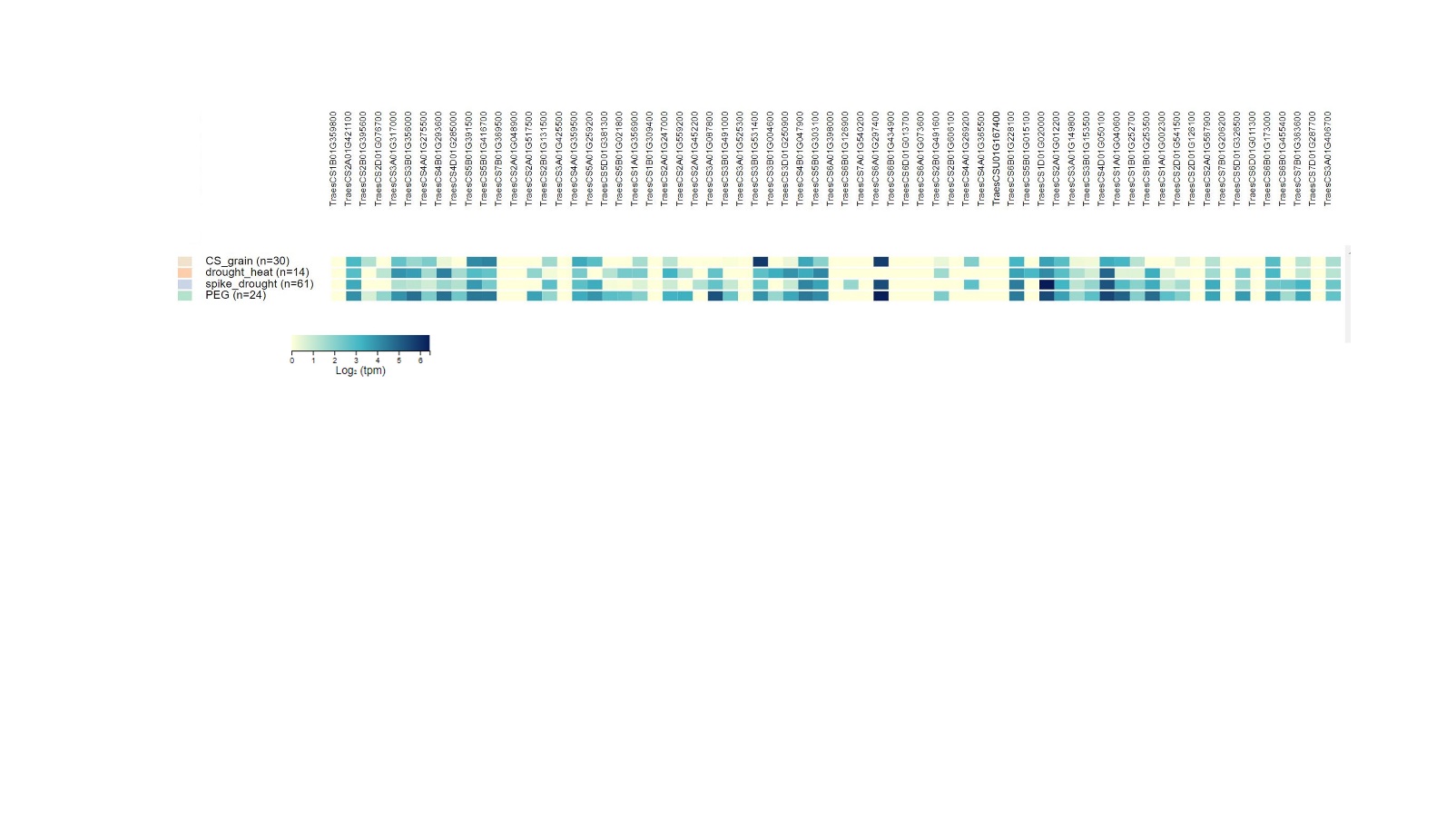
**

**Figure S3**. In silico expression prediction in drought stress condition for MTA associated most proximal genes.
